# CDSeq: A novel complete deconvolution method for dissecting heterogeneous samples using gene expression data

**DOI:** 10.1371/journal.pcbi.1007510

**Published:** 2019-12-02

**Authors:** Kai Kang, Qian Meng, Igor Shats, David M. Umbach, Melissa Li, Yuanyuan Li, Xiaoling Li, Leping Li

**Affiliations:** 1 Biostatistics and Computational Biology Branch, National Institute of Environmental Health Sciences, Durham, North Carolina, United States of America; 2 Signal Transduction Laboratory, National Institute of Environmental Health Sciences, Durham, North Carolina, United States of America; National Center for Biotechnology Information (NCBI), UNITED STATES

## Abstract

Quantifying cell-type proportions and their corresponding gene expression profiles in tissue samples would enhance understanding of the contributions of individual cell types to the physiological states of the tissue. Current approaches that address tissue heterogeneity have drawbacks. Experimental techniques, such as fluorescence-activated cell sorting, and single cell RNA sequencing are expensive. Computational approaches that use expression data from heterogeneous samples are promising, but most of the current methods estimate either cell-type proportions or cell-type-specific expression profiles by requiring the other as input. Although such partial deconvolution methods have been successfully applied to tumor samples, the additional input required may be unavailable. We introduce a novel complete deconvolution method, CDSeq, that uses only RNA-Seq data from bulk tissue samples to simultaneously estimate both cell-type proportions and cell-type-specific expression profiles. Using several synthetic and real experimental datasets with known cell-type composition and cell-type-specific expression profiles, we compared CDSeq’s complete deconvolution performance with seven other established deconvolution methods. Complete deconvolution using CDSeq represents a substantial technical advance over partial deconvolution approaches and will be useful for studying cell mixtures in tissue samples. CDSeq is available at GitHub repository (MATLAB and Octave code): https://github.com/kkang7/CDSeq.

## Author summary

Understanding the cellular composition of bulk tissues is critical to investigate the underlying mechanisms of many biological processes. Single cell sequencing is a promising technique, however, it is expensive and the analysis of single cell data is non-trivial. Therefore, tissue samples are still routinely processed in bulk. To estimate cell-type composition using bulk gene expression data, computational deconvolution methods are needed. Many deconvolution methods have been proposed, however, they often estimate only cell type proportions using a reference cell type gene expression profile, which in many cases may not be available. We present a novel complete deconvolution method that uses only bulk gene expression data to simultaneously estimate cell-type-specific gene expression profiles and sample-specific cell-type proportions. We showed that, using multiple RNA-Seq and microarray datasets where the cell-type composition was previously known, our method could accurately determine the cell-type composition. By providing a method that requires a single input to determine both cell-type proportion and cell-type-specific expression profiles, we expect that our method will be beneficial to biologists and facilitate the research and identification of mechanisms underlying many biological processes.

This is a *PLOS Computational Biology* Methods paper.

## Introduction

The measured expression of a gene in a bulk sample reflects the expression of that gene in every cell in the sample. Consequently, the measured gene expression profile (GEP) of a tissue sample is commonly regarded as a weighted average of the GEPs of the different component cell types [1, 2].

The heterogeneous nature of bulk tissue samples complicates the interpretation of bulk measurements such as RNA-Seq. Often researchers are interested in understanding whether an experimental treatment targets one particular cell type in a heterogeneous tissue or in investigating possible sources of variation among samples [3]. For example, the composition of tumor-infiltrating lymphocytes impacts tumor growth and patients’ clinical outcomes [4–9]. With expression measurements on bulk tissue, it is often difficult to distinguish between low expression in a highly abundant cell type and high expression in less abundant cell type [3]. Consequently, understanding the cell-type composition of each sample and the GEP of each constituent cell type becomes important. “Deconvolution” is a generic term for a procedure that estimates the proportion of each cell type in a bulk sample together with their corresponding cell-type-specific GEPs [10, 11]. Deconvolution can be approached experimentally using flow cytometry or single cell RNA sequencing. For solid tissues, these techniques require isolating individual cells, thereby presenting laboratory challenges as well as potentially sacrificing a systems perspective. Single cell RNA sequencing is also expensive and requires challenging data handling and analysis [12, 13].

Deconvolution can also be approached computationally using GEP profiles from collections of bulk tissue samples [11, 14]. Many deconvolution methods have been developed in the past decade. The pioneering work of Venet et al. [15] employed an algorithm based on matrix factorization to deconvolve a matrix of GEPs (each normalized to sum to 1) into a product of two matrices, one containing the cell-type proportions for each sample and the other containing the GEPs for each cell type. The constraints required for each matrix in the product (proportions must be nonnegative and sum to 1 across cell types; expression levels must obey the same constraints across genes) impose technical challenges on matrix factorization in this context. Deconvolution methods that are based on nonnegative matrix factorization (NMF) may not be guaranteed to find cell-type-specific components [16, 17]. Consequently, most existing methods only perform partial deconvolution: either the algorithms require cell-type proportions as input to estimate cell-type-specific GEPs [1, 17–20] or vice versa [21–29]. These methods generally use regression techniques and some also use marker genes [3, 30, 31] to estimate the unknowns of interest. Such deconvolution approaches have shown important findings [7, 31], however they could suffer if the needed information is unavailable or if the fidelity of reference GEP profiles or cell-type proportions is questionable.

Our goal was to develop a complete deconvolution method using only bulk RNA-Seq data by estimating cell-type proportions and cell-type-specific GEPs simultaneously. The underlying model was based on latent Dirichlet allocation (LDA) [32], a probabilistic model designed for natural language processing. LDA was designed to use text corpora as input and extract essential structure, namely, the topics that constitute the content of documents in the corpus. The problem of deriving abstract yet meaningful topics from a corpus of documents shares a fundamental similarity with the problem of extracting cell-type-specific information from bulk RNA-Seq data. The original LDA model cannot, however, fully capture the complexity of bulk RNA-Seq data. Although some existing methods are based on the LDA model [26, 27, 33], those methods were designed for partial deconvolution and require cell-type-specific GEPs as input. We refer to our method as CDSeq (Complete Deconvolution for Sequencing data). We assessed CDSeq’s performance using several synthetic and real experimental datasets with known cell-type composition and cell-type-specific GEPs and compared it with seven other deconvolution methods.

## Materials and methods

### Overview of CDSeq

**Fig 1:**
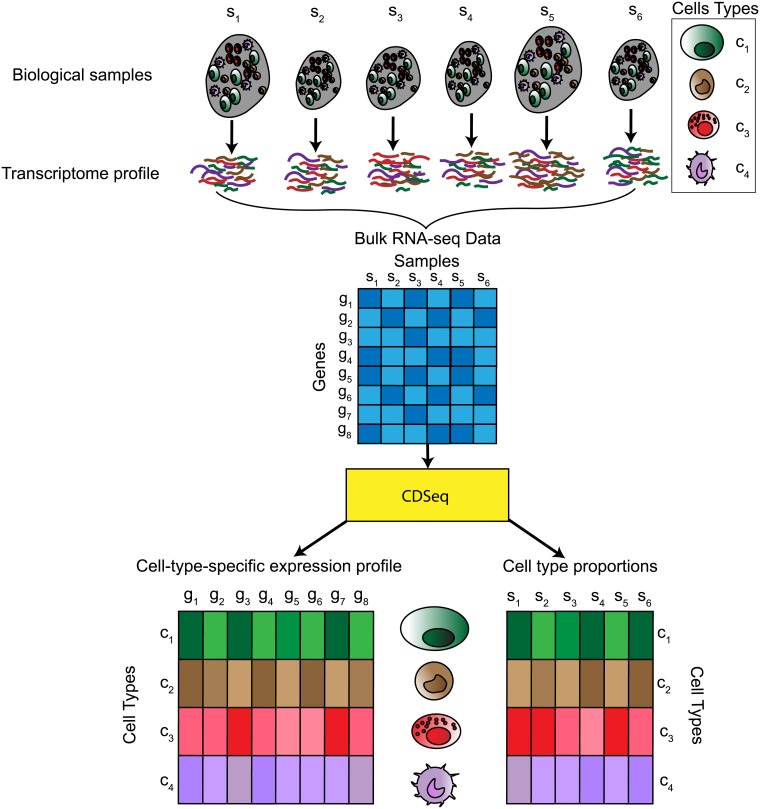
Schematic of the CDSeq approach. Heterogeneous samples consist of different cell types. The bulk RNA-Seq profile represents a weighted average of the expression profiles of the constituent cell types. CDSeq takes as input the bulk RNA-Seq data for a collection of samples and performs complete deconvolution that outputs estimates of both the cell-type-specific expression profiles and the cell-type proportions for each sample. This Figure depicts a simple scenario of six biological samples comprising four cell types, each with gene expression measurements on eight genes.

Using only bulk RNA-Seq expression data for multiple samples as input, CDSeq provides estimates of both cell-type-specific GEPs and sample-specific cell-type proportions simultaneously (Fig 1). Our model extends the LDA model in the following ways: first, the random variable that models cell-type-specific GEPs depends on gene length [34]; second, the probability of having a read from a cell type depends on both the proportion of that cell type present in a sample and the typical amount of RNA produced by cells of that type [24, 35]. This second extension accommodates the possibility that different cell types produce different amounts of RNA, a circumstance that could bias estimates of cell-type proportions.

To describe our model and the statistical inference scheme, we first introduce the notation. Let *M* denote the number of samples and *T* denote the number of cell types comprising each heterogeneous sample. We model the vector containing the cell-type-specific proportions for sample *i*, denoted *θ*_*i*_ = (*θ*_*i*,1_, ⋯, *θ*_*i*,*T*_) ∈ *S*^*T*^, where *S*^*T*^ denotes a (*T* − 1)-simplex, as a Dirichlet random variable with hyperparameter $\alpha =\left(\alpha_{1},\cdots ,\alpha_{T}\right)\in R_{+}^{T}$. Next, let *G* denote the number of genes in the reference genome to which reads are mapped. We denote the GEP of pure cell type *t*, a vector of gene expression values for the entire genome normalized to sum to 1, as *ϕ*_*t*_ = (*ϕ*_*t*,1_, ⋯, *ϕ*_*t*,*G*_) ∈ *S*^*G*^, where *S*^*G*^ denotes a (*G* − 1)-simplex and model it as a Dirichlet random variable with hyperparameter $\beta =\left(\beta_{1},\cdots ,\beta_{G}\right)\in R_{+}^{G}$. With *T* cell types in all *M* samples, the matrices *θ* = [*θ*_1_, ⋯, *θ*_*M*_] and *ϕ* = [*ϕ*_1_, ⋯, *ϕ*_*T*_] encapsulate all the features that we seek to estimate from the data based on our model.

We denote the true GEP of heterogeneous sample *i* by Φ_*i*_ = (Φ_*i*,1_, Φ_*i*,2_, Φ_*i*,*G*_) ∈ *S*^*G*^. Φ_*i*_ is a weighted average of the pure cell-type GEPs with weights given by the sample-specific cell-type proportions, namely, $\Phi_{i}=\sum_{t=1}^{T}\theta_{i,t}\phi_{t}$. This random variable controls the rate of generating RNA copies from genes.

We do not observe the true Φ_*i*_ directly but instead observe reads from each sample and we can obtain the read assignments to genes. Assume that the length of every sequenced read, denoted *m*, is the same. Let categorical random variable *r*_*i*,*j*_ denote read *j* from sample *i* (after mapped to a gene, the possible outcomes of *r*_*i*,*j*_ depend on the gene and its length), and let categorical random variable *g*_*i*,*j*_ ∈ {1, ⋯, *G*} denote the gene or transcript assignment of read *r*_*i*,*j*_. Both ${\left\{r_{i,j}\right\}}_{i=1,j=1}^{M,N_{i}}$ and ${\left\{g_{i,j}\right\}}_{i=1,j=1}^{M,N_{i}}$ are observed for every heterogeneous sample, where *N*_*i*_ denotes the number of reads from sample *i*. In transcript *k*, the number of positions in which a read can start is ${\tilde{\ell }}_{k}=\ell_{k}-m+1$ where *ℓ*_*k*_ is the length of transcript *k*. The adjusted length ${\tilde{\ell }}_{k}$ is called the effective length of transcript *k*, then *r*_*i*,*j*_ has ${\tilde{\ell }}_{g_{i,j}}$ possibilities [34]. If the reads are mapped to genes instead of to transcript isoforms, then we need to consider the effective length of gene, denoted by *ℓ*_*g*_, which is total length of all the transcripts comprising the gene after projection into genomic coordinates. All the analyses reported here were done on the gene level.

Different cell types may generate different amounts of RNA owing to their varying sizes, therefore we employ a Poisson random variable with parameter *η*_*t*_ to model the number of reads generated from cell type *t*. Let *η* = (*η*_1_, ⋯, *η*_*T*_). Parameter *η* can be estimated from RNA-Seq read counts from pure cell types using the unweighted sample mean, a maximum likelihood unbiased estimator. CDSeq uses the user-specified *η* to adjust RNA proportions to cell proportions. If such information is not provided, CDSeq will assign each element of *η* the same value, indicating no differences in cell sizes (*θ* then represents RNA proportions, not cell proportions).

Finally, to complete specification of our model, we need to be able to assign reads in the heterogeneous sample to individual cell types; thus, we introduce a latent categorical random variable *c*_*i*,*j*_ ∈ {1, ⋯, *T*} that is the cell type indicator of read *r*_*i*,*j*_. Our model specifies that RNA-Seq reads from bulk tissues are generated as follows:

1. Generate gene expression profiles for different cell types, i.e., *ϕ*_*t*_ ∼ *Dir*(*β*) for cell type *t*, *t* = 1, ⋯, *T*, and $\phi_{t}\in R^{G}$.
2. Choose *θ*_*i*_ ∼ *Dir*(*α*) which denotes the mixture proportion of different cell types in the sample *i*, *i* = 1, ⋯, *M*, and $\theta_{i}\in R^{T}$.
3. For each of the *N*_*i*_ RNA-Seq reads in sample *i*, where *N*_*i*_ denotes the total reads of sample *i*
  - Choose a cell type $c_{i,j}∼\text{Categorical}({\tilde{\theta }}_{i})$, where *j* = 1, ⋯, *N*_*i*_, $c_{i,j}\in R$, ${\tilde{\theta }}_{i}\propto \theta_{i}\cdot \eta$ (adjusting cell proportion *θ* to RNA proportion $\tilde{\theta }$) and “·” denotes element-wise product.
  - Choose a gene $g_{i,j}∼\text{Categorical}({\tilde{\phi }}_{c_{i,j}})$, where *j* = 1, ⋯, *N*_*i*_, $g_{i,j}\in R$, ${\tilde{\phi }}_{c_{i,j}}\propto \phi_{c_{i,j}}\cdot \tilde{\ell }$ (adjusting gene expression $\phi_{c_{i,j}}$ by considering the effective gene length) and ${\tilde{\phi }}_{c_{i,j}},\phi_{c_{i,j}},\tilde{\ell }\in R^{G}$. $\tilde{\ell }$ denotes the effective lengths of genes and “·” denotes element-wise product.
  - Generate a read sequence *r*_*i*,*j*_ by uniformly choosing one of the ${\tilde{\ell }}_{g_{i,j}}$ positions in gene *g*_*i*,*j*_.

**Fig 2:**
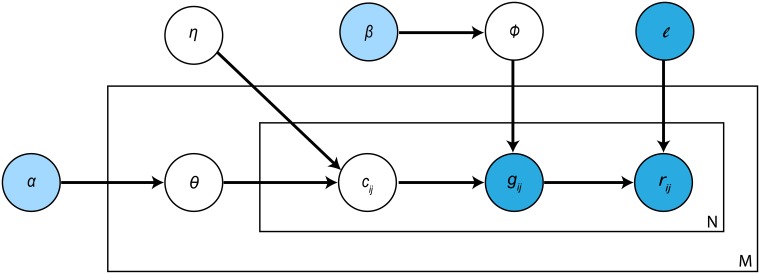
Graphical representation of CDSeq probabilistic model. The light blue nodes, *α*, *β*, denote the hyperparameters that are assumed to be known. The dark blue nodes, *ℓ*, *g*_*ij*_, *r*_*ij*_, denote the values of observable random variables (either measured in the study or established in previous studies) whereas the white nodes, *η*, Φ, *θ*, *c*_*ij*_, are unobservable random variables that need to be inferred from data. The outer box represents samples where *M* is the sample size, and the inner box denotes the RNA-Seq data of a sample where *N* is the total number of reads from the sample (see S1 Methods for details).

To this end, a graphical model of CDSeq is presented in Fig 2 depicting the stochastic process of generating RNA-Seq data. Details on parameter estimation and method for determining the optimal number of cell types in the data are provided in S1 Methods.

The cell types delineated by CDSeq are mathematical entities that must be matched to corresponding biological cell types. To match the CDSeq cell types to actual cell types requires a list of reference cell-type-specific GEPs and metric of similarity (for example, Pearson’s correlation coefficient or Kullback-Leibler divergence) (S1 Methods). Depending on the application, many reference profiles are available, e.g., the LM22 [25] for immune cell subsets. We employed the Munkres algorithm [36] in CDSeq for cell type association when a list of reference GEPs is provided. An alternative way to identify CDSeq-estimated cell types, without using a reference GEP profile, is to evaluate enrichment scores of marker gene sets similar to scRNA-Seq analysis [9].

### Computational complexity and a data dilution strategy to speed up the algorithm

In CDSeq, the Gibbs sampler iteratively assigns a cell type to each read using a binary search with a time complexity of O(logT), where *T* is the number of cell types. Assume the number of total reads is *R*, then the time complexity of the Gibbs sampler is O(RlogT). If needed, we have also provided a way to speed up the CDSeq using a data dilution strategy (S1 Methods). Specifically, one could divide all the read counts by a positive constant—dilution factor. We systematically tested the effect of the dilution factor on the accuracy of estimation using both synthetic and experimental mixture data. For our 32 experimental mixtures with ∼ 20*k* genes using a dilution factor of 10, it took CDSeq about 2 hours to finish on an iMac (3.5 GHz Intel Core i7 with 32GB memory).

### A quasi-unsupervised learning strategy

CDSeq is an unsupervised learning method that aims at discovering the latent pattern from data without any labeling or prior knowledge. The GEPs of the cell types identified by CDSeq may not closely match any available pure cell line GEPs. This issue may arise because highly correlated GEPs of multiple cell types or subtypes complicates the deconvolution problem and renders CDSeq less able to definitively separate cell types. For example, this issue is escalated in the problem of deep deconvolution. Deep deconvolution refers to the problem of using a whole blood or peripheral blood mononuclear cell (PBMC) sample to estimate the proportions and gene expression profiles of a greater number of cell subtypes, going further down into the hematopoietic tree [11]. To mitigate this kind of problem, we developed a quasi-unsupervised learning strategy. The idea is to provide CDSeq some guidance that leads the algorithm to more biologically meaningful latent information. The guidance consists of appending a set GEPs of pure cell lines to the original input GEPs of heterogeneous samples. The choice of GEPs appended should reflect pure cell lines that are believed to constitute the samples.

To apply the quasi-unsupervised approach, one could simply append a set of pure cell line GEPs to the GEPs of the bulk samples for the same genes. Each appended pure cell line GEP is treated as a “bulk” sample by CDSeq. For example, let *X*_*G*×*M*_ denote bulk RNA-Seq data for *G* genes and *M* samples, to append a set of *W* pure cell line GEPs, say, ${\tilde{X}}_{G\times W}$, one would need to create $Y_{G\times (M+W)}=\left[X_{G\times M},{\tilde{X}}_{G\times W}\right]$, a data matrix with *G* rows (genes) and *M* + *W* columns (samples), as the input for CDSeq. We showed that, using this quasi-unsupervised strategy, CDSeq provided more informative estimates than those obtained using the fully unsupervised mode (Results). We call this learning strategy “quasi-unsupervised” because, although we do not incorporate any labeling information within CDSeq algorithm itself, we do inject strong signals about likely relevant cell types into the input data. In short, CDSeq is not explicitly aware of such labeling information (pure cell line GEPs appended to input) and treats them the same as other input samples unlike traditional semi-supervised methods where the labeling information is explicitly taken into account by the algorithms.

### Comparisons with other deconvolution methods

We compared CDSeq to seven competing deconvolution methods using their default settings when applicable (Table 1). We present detailed comparisons with csSAM and CIBERSORT in the main text and full comparisons in S1–S8 Figs. For the purpose of comparison, we used reads per kilobase per million mapped reads (RPKM) [37] normalization as input for RNA-Seq data. Using our experimental mixtures, we also showed that the RPKM-normalized RNA-Seq data fit well with the linearity assumption employed by deconvolution methods (S10 Fig). Details on the linearity assumption are given in S1 Methods.

**Table 1:** Deconvolution methods for comparison.

Deconvolution methods	Estimate proportions	Estimate GEPs	Reference	Dataset*
CDSeq	✓	✓		①-⑥
CIBERSORT	✓		[25]	①-⑥
DeconRNAseq	✓		[22]	①-⑥
UNDO	✓		[29]	①-②
csSAM		✓	[1]	①-③
DSA		✓	[20]	①-③
deconf	✓	✓	[16]	①-⑥
ssKL	✓	✓	[17]	①-⑥

* **Dataset**: ① Synthetic mixtures (S1 Table); ② Experimental mixtures (S2 Table); ③ Mixtures of liver, lung and brain [1]; ④ Leukocyte subtypes(LM22) [25]; ⑤ Lymphoma samples [25]; ⑥ PBMC samples [25].

### Synthetic and experimental mixtures and gene expression profiling

We generated 40 synthetic samples (S1 Table) and 32 experimental mixtures measured using RNA-Seq (S2 Table) for benchmarking CDSeq. The details of data generation procedure are given in S1 Methods.

## Results

### Performance on synthetic data

We first benchmarked CDSeq on synthetic mixtures with known compositions that we created numerically from publicly available GEPs from Cold Spring Harbor Laboratory. In this synthetic numerical experiment, we amplified the potential bias between RNA proportions and cell-type proportions by artificially increasing the RNA amount of certain cell types before mixing them together to generate the synthetic samples. We generated 40 synthetic samples where each sample was a combination of six different cell types in different proportions (S1 Table).

**Fig 3:**
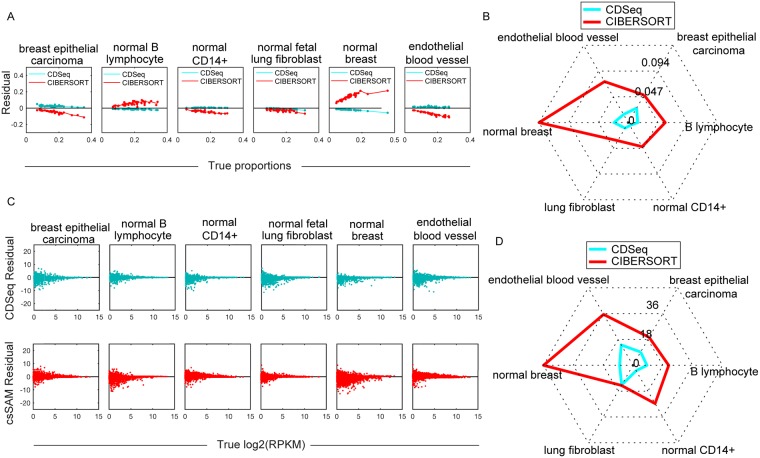
Deconvolution of synthetic mixtures. We ran CDSeq with six cell types, *α* = 5, *β* = 0.5, and 700 MCMC runs. (A) Difference (“residual”) between estimated and true cell-type proportion plotted against true proportion for CDSeq (green) and CIBERSORT (red). Each plotted point represents the value for a single sample. (B) Radar plot of RMSE for estimates of sample-specific cell-type proportions. CDSeq (green); CIBERSORT (red). (C) Difference (“residual”) between estimated and true log2 gene expression level (log2(RPKM)) plotted against true log2 gene expression level for CDseq (green) and csSAM (red). Each plotted point represents a single gene, 22498 genes total. (D) Radar plot of RMSE for gene expression levels (RPKM). CDSeq (green); csSAM (red).

In estimating cell-type proportions, CDSeq outperformed CIBERSORT, showing smaller differences between the true and estimated proportions for each cell type and, consequently, smaller root mean square error (RMSE) (Fig 3 and S1 Fig). The RMSE of CDSeq was overall 77% lower than that of CIBERSORT.

**Fig 4:**
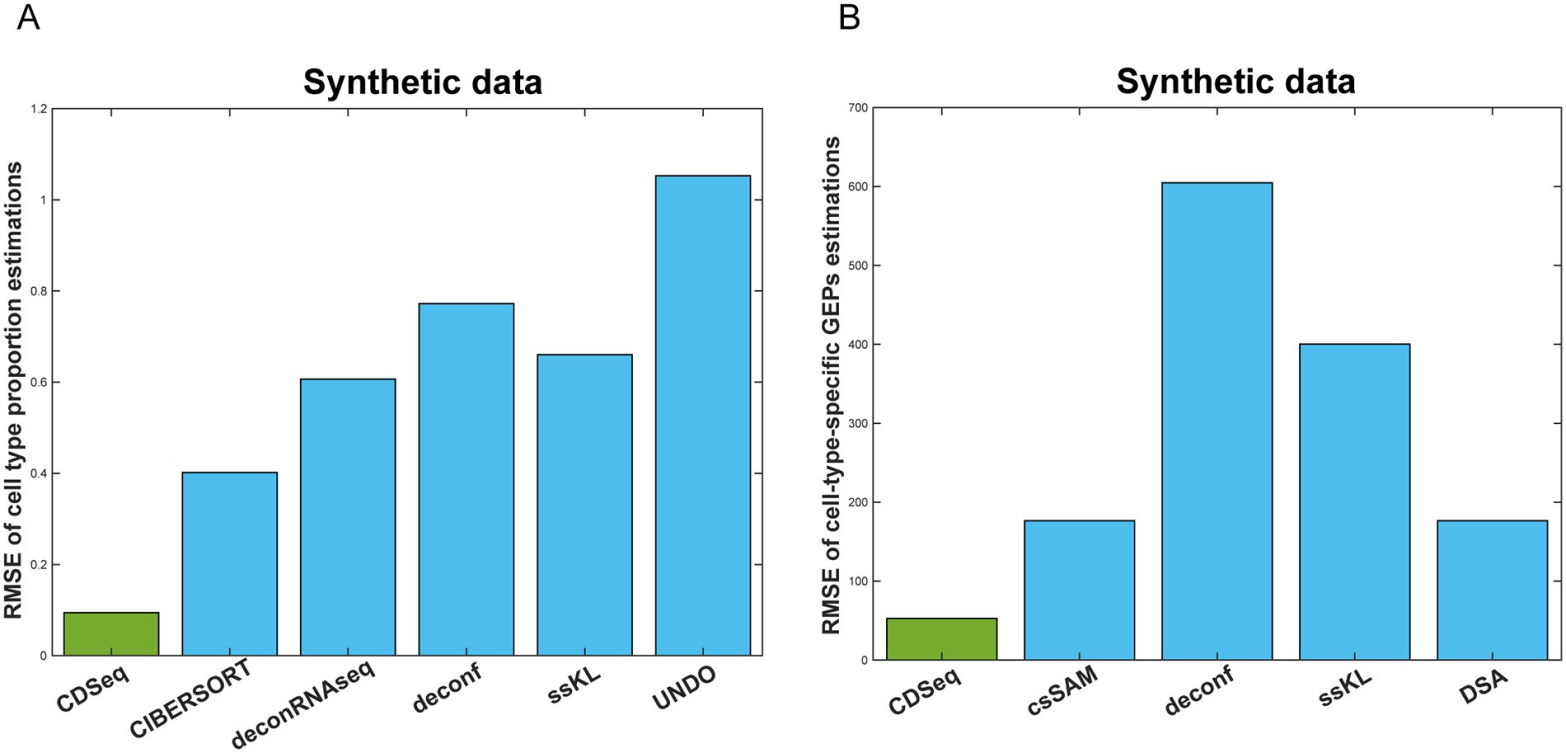
Performance comparisons on synthetic mixtures. (A) RMSEs of sample-specific cell-type proportion estimations; (B) RMSEs of cell-type-specific GEPs estimations.

In estimating GEPs, performances of CDSeq and csSAM were comparable. However, CDSeq still outperformed csSAM with 64% lower RMSE values than csSAM (Fig 3 and S1 Fig). Notice that RMSE is not calculated on log scale because some of the gene expression values are zeros. In addition, CDSeq outperformed all other seven competing deconvolution methods as shown in Fig 4. CDSeq in general requires more running time than competing methods. For the synthetic mixtures, CDSeq took about 2 hours and CIBERSORT took about 3 hours whereas the remaining tools took seconds to complete.

### Performance on mixture of RNAs extracted from cultured cells

**Fig 5:**
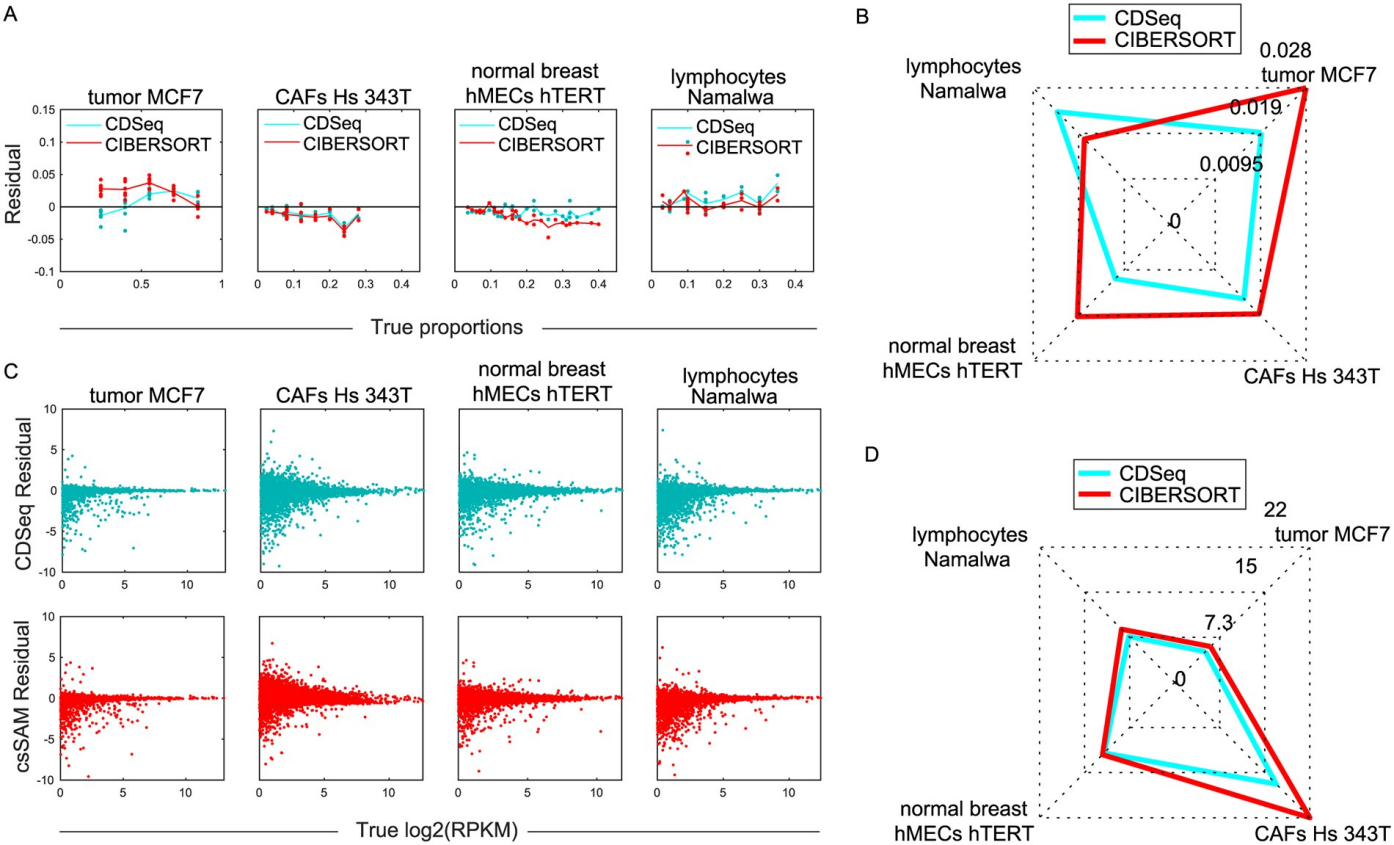
Deconvolution of mixed RNA from cultured cell lines. We ran CDSeq with four cell types, *α* = 5, *β* = 0.5, and 700 MCMC runs. (A) Difference (“residual”) between estimated and true cell-type proportion plotted against true proportion for CDSeq (green) and CIBERSORT (red). Each plotted point represents the value for a single sample. (B) Radar plot of RMSE for estimates of sample-specific cell-type proportions. CDSeq (green); CIBERSORT (red). Total RMSE summing over cell types is 17% smaller for CDseq compared to CIBERSORT. (C) Difference (“residual”) between estimated and true log2 gene expression level (log2(RPMK)) plotted against true log2 gene expression level for CDseq (green) and csSAM (red). Each plotted point displays the expression value of a single gene, 19653 genes in total. (D) Radar plot of RMSE for gene expression levels. CDSeq (green); csSAM (red). Total RMSE of gene expression (summing over cell types) is 16% smaller for CDseq compared to csSAM.

**Fig 6:**
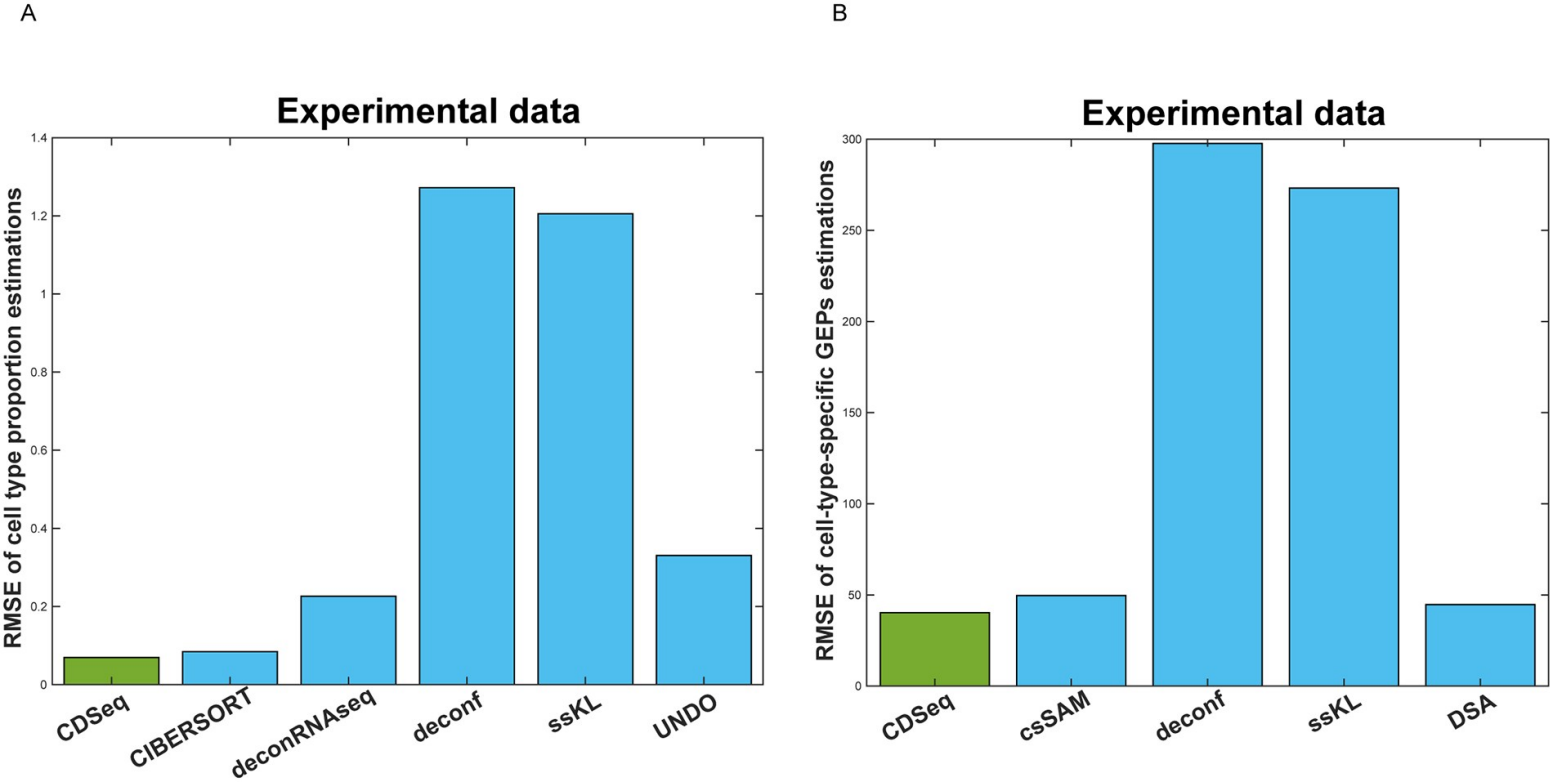
Performance comparisons on experimental mixtures. (A) RMSEs of sample-specific cell-type proportion estimations; (B) RMSEs of cell-type-specific GEPs estimations.

Our second performance evaluation used data from a designed experiment that created 32 mixture samples using known RNA proportions isolated from four pure cell lines (S1 Methods). CDSeq predicted both the cell proportions and GEPs well (Fig 5). CDSeq generally outperformed all competitors as indicated by smaller total RMSE (Fig 6); For example, CDSeq had 17% smaller RMSE than CIBERSORT for estimating cell-type proportions and 16% smaller RMSE than csSAM for estimating GEPs (S2 Fig).

### Dissecting mixtures of liver, lung, and brain cells

**Fig 7:**
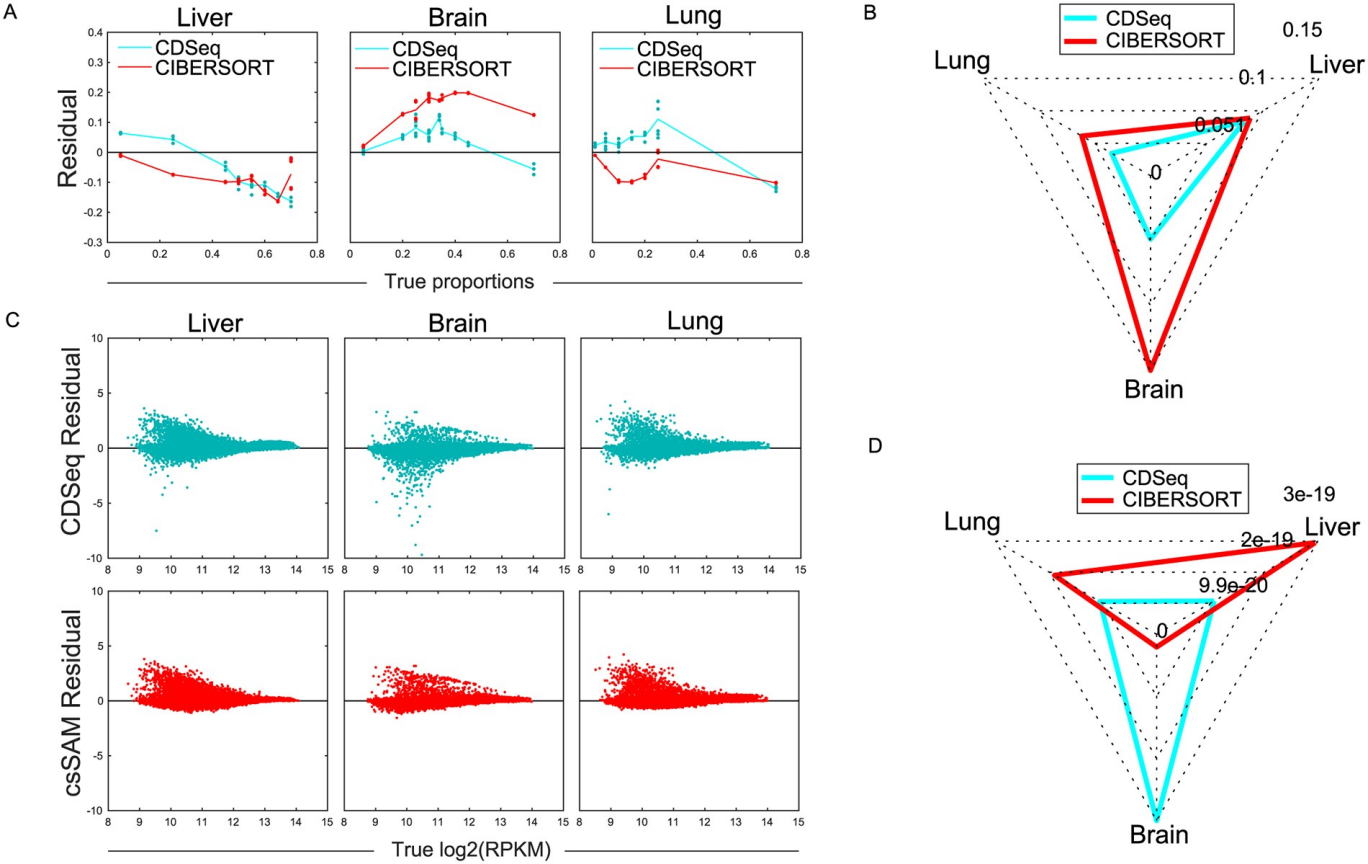
Deconvolution of mixed liver, lung and brain cell lines. Comparisons with CIBERSORT and csSAM on mixtures of liver, brain and lung cells. (A) Residual of proportion estimation; (B) Radar plot of RMSE for proportion estimation; (C) Residual of GEPs estimation; (D) Radar plot of RMSE for GEPs estimation.

We evaluated CDSeq using the experimental data set designed for csSAM [1]. The microarray data set consists of 11 mixtures (each with 3 replicates) of liver, brain and lung cells with varying known RNA proportions. We showed that CDSeq outperformed all competing methods in estimating both cell-type-specific GEPs and sample-specific proportions of cell types (Fig 7 and S3 Fig). For example, the RMSE of the CDSeq-estimated cell proportion was 44% lower than the corresponding CIBERSORT RMSE, and the RMSE of CDSeq-estimated GEPs was similar to the corresponding csSAM RMSE.

### Evaluation using leukocyte subtypes

To test the performance of CDSeq on some extreme cases, we applied CDSeq to a set of GEPs from pure cell lines. We chose LM22 designed by Newman et al. [25], which comprises 22 human hematopoietic cell phenotypes. Thus, the GEPs of some of the cell lines are highly correlated with each other. CDSeq successfully uncovered the 22 cell types. CDSeq’s estimates of cell-type proportions, which should be 100% for these pure cell lines, generally exceeded 90%. Overall, CDSeq performed comparably with CIBERSORT, deconRNAseq, and ssKL in estimating sample-specific cell-type proportions (S4 Fig), even though CIBERSORT and deconRNAseq require the GEPs of leukocyte subtypes as input (deconf performed much worse, in comparison to CDSeq). For cell-type-specific GEPs estimation, CDSeq performed comparably with deconf and ssKL (S4 Fig).

### Immune cell analysis of lymphoma data with comparison to flow-cytometry

**Fig 8:**
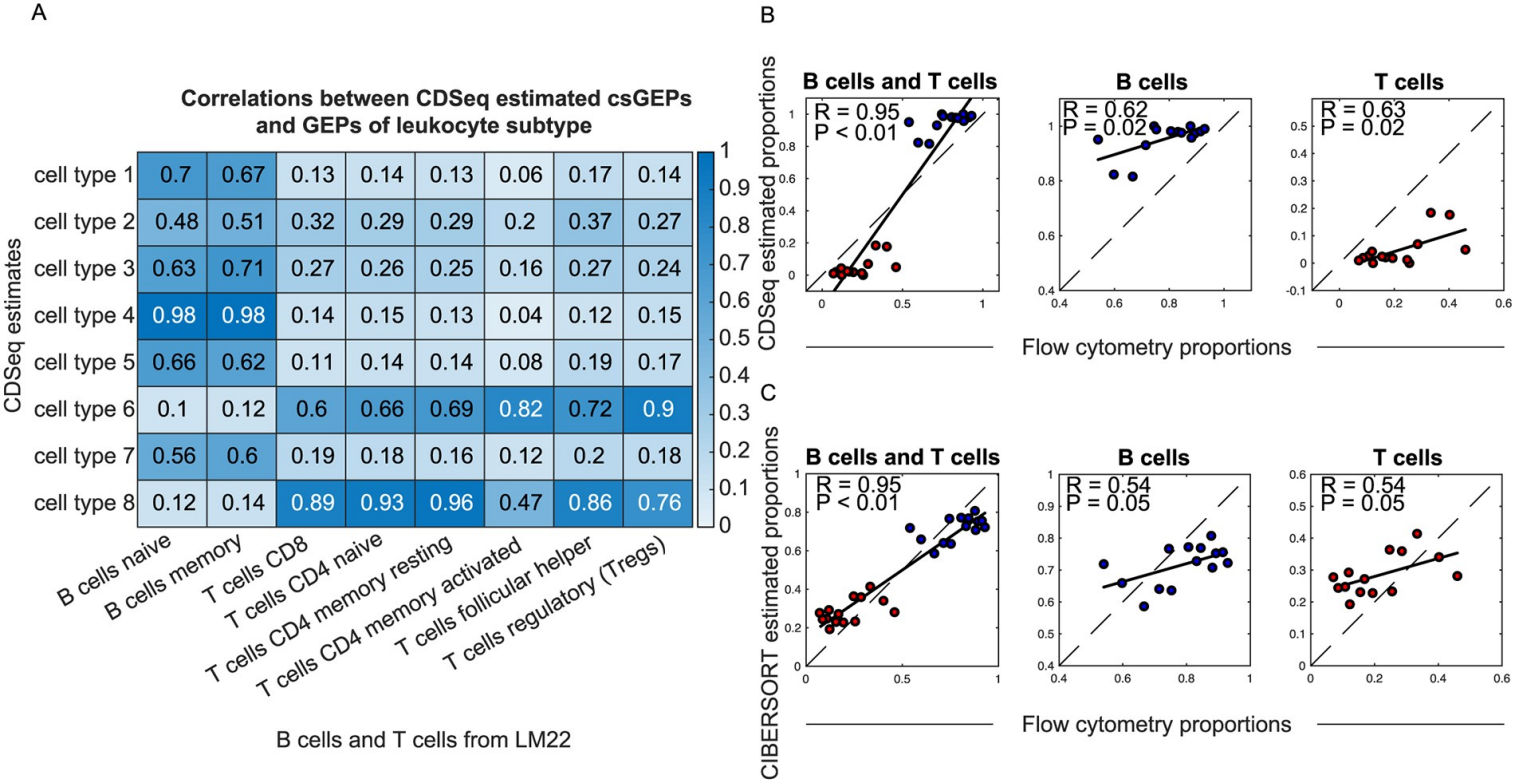
Comparison of CDSeq using the quasi-unsupervised strategy with CIBERSOFT on deconvolution of B cells and T cells in lymphoma samples. We ran CDSeq with 22 cell types, *α* = 0.5, *β* = 0.5, and 700 MCMC runs. We considered an anonymous CDSeq-identified cell type to match one of the B cell (blue dots) or T cell subtypes (red dots) if the Pearson correlation of their GEPs exceeded 0.6. (A) Correlation between estimated GEPs and true GEPs; (B) CDSeq estimated proportions versus flow cytometry; (C) CIBERSORT estimation versus flow cytometry.

We evaluated CDSeq against flow-cytometry measurements of leukocyte content in solid tumors. Data comprised GEPs from 14 bulk follicular lymphoma samples and corresponding flow-cytometry measurements [25]. Our goal was to estimate the proportions of B cells (naive B cell and memory B cell) and T cells (CD8 T cell, CD4 naive T cell, CD4 memory resting T cell, CD4 memory activated T cell, follicular helper T cell, regulatory T cell) in those 14 samples using CDSeq. We set the number of cell types to be eight (the number of all B cell and T cell subtypes in our reference file). We carried out two approaches—fully unsupervised and quasi-unsupervised. The quasi-unsupervised performed better than the fully unsupervised approach for this dataset when the GEPs of constituent cell types are highly correlated (S6 and S7 Figs). We showed that CDSeq outperformed deconf and ssKL and performed comparably with CIBERSORT and DeconRNAseq (Fig 8 and S7 Fig), both of which require a reference GEP set as input.

### CDSeq on deep deconvolution

To assess CDSeq’s performance on deep deconvolution, we used a set of 20 PBMC samples [25]. To evaluate performance, we also used information provided by Newman et al. [25]: namely, flow-cytometry measurements for nine of the 22 leukocyte subtypes (the only subtypes with flow cytometry available). That LM22-provided GEPs of about half of these nine subtypes were highly correlated (S5 Fig) should challenge CDSeq’s ability both to find the corresponding GEPs of those nine subtypes in the 20 PMBC samples and to accurately estimate their proportions. We first ran CDSeq in fully unsupervised mode and set the number of cell types to be 22. Using the GEPs of LM22 as references, we found that CDSeq could not uncover the nine subtypes (S8 Fig), possibly because of the highly correlated GEPs among subtypes.

**Fig 9:**
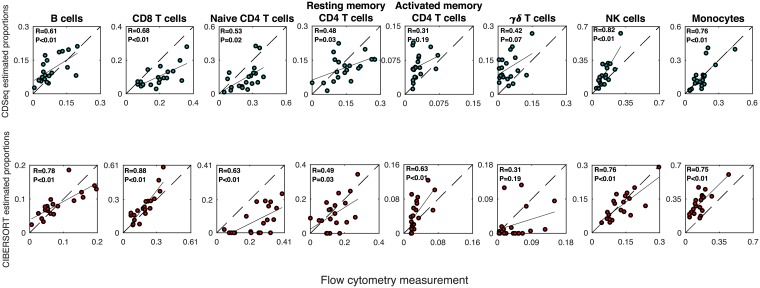
Deep deconvolution of PBMC data. We applied CDSeq using the quasi-unsupervised learning strategy and ran CDSeq with 22 cell types, *α* = 50, *β* = 20. The black line is the linear regression line; the dashed line is the *x* = *y* line; *R* is the correlation coefficient; and *P* is the p-value for testing the null hypothesis of no correlation.

To improve estimation, we turned to the quasi-unsupervised strategy when running CDSeq by appending the 22 GEPs of LM22 to the 20 samples, 42 samples in total. Using the 0.6 correlation threshold to match CDSeq-identified cell types to the corresponding 22 leukocyte subtypes, we found that the quasi-unsupervised strategy improved CDSeq’s performance (Fig 9 and S8 Fig): one CDSeq-identified cell type matched both naive and activated B cells; another matched both resting and activated mast cells; two CDSeq-identified cell types did not match any of the 22 LM22 known subtypes; the remainder matched only one LM22 subtype each.

We next compared CDSeq-estimated cell-type proportions of these nine cell subtypes to flow-cytometry measurements. However, since CDSeq could not distinguish between naive B cells and memory B cells, we combined these two types into one overall B cell type, resulting in eight total subtypes (Fig 9 and S8 Fig). In restricting attention to the resulting eight subtypes, we renormalized their proportions to sum to one for comparison with corresponding flow cytometry measured proportions.

For six of the eight subtypes, the CDSeq-estimated relative proportions were significantly correlated (*p* < 0.05) with the flow-cytometry-based relative proportions. The correlations with activated memory CD4 and *γδ* T cells were not significant (*p* = 0.31 and 0.07, respectively). The CIBERSORT estimated relative proportions were significant correlated (*p* < 0.05) with the corresponding flow-cytometry-based relative proportions for all subtypes except *γδ* T cells (*p* = 0.19). In an overall comparison of CDSeq and CIBERSORT estimates, however, the total RMSE of CDSeq was about 6% lower than that of CIBERSORT. On the other hand, the estimated relative proportions by both CDSeq and CIBERSORT showed systematic bias in departing from equality with the flow-cytometry-based proportions. Besides the possible technical issues of flow-cytometry and the fidelity of the LM22 reference profiles, another possible reason for this systematic bias with this microarray data is that flow cytometry reports relative cell proportions whereas CDSeq and CIBERSORT report relative RNA proportions. Though CDSeq is capable of reporting either RNA proportions or cell proportions from RNA-Seq raw counts, it can report only RNA proportions with microarray data. We show CDSeq outperformed all other competing methods by having the smallest RMSE in S8 Fig.

### Estimating the number of cell types present from the data

We have been applying CDSeq by fixing the number of cell types at the correct number, since we know it in advance. CDSeq can, however, estimate the number of constituent cell types in a collection of samples, if necessary, by maximizing the posterior distribution (S1 Methods). The framework of CDSeq is built for RNA-Seq raw count data, therefore, raw count data is required for estimating the number of cell types. Consequently, we did not apply this feature for microarray data.

**Fig 10:**
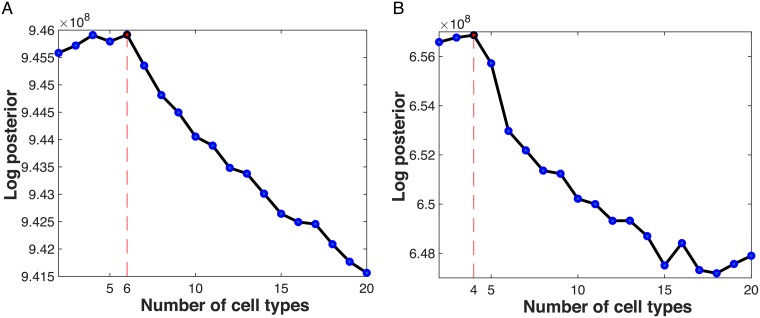
Estimating the number of cell types. The maximum of the log posterior provides an estimate of the number of cell types. (A) synthetic data; (B) mixed RNA data. In each data set, the method correctly estimated the number of cell types.

Applying this method to the synthetic data and to the data on mixed RNA described above correctly estimated number of cell types in each case (Fig 10). In Fig 10(A), the values of log posterior at 4 and 6 cell types are close, however, the maximum occurs at 6.

## Discussion

As a complete deconvlution method, CDSeq has many advantages over existing partial deconvolution methods, like csSAM [1] and CIBERSORT [25]. For example, CDSeq requires only one input (expression data from mixtures) to produce two outputs (estimates of cell-type-specific GEPs and sample-specific cell-type proportions). Partial deconvlution methods that require cell-type-specific GEPs as input face concerns about the accuracy or appropriateness of the reference profiles. Complete deconvolution avoids these concerns, although reference GEPs or marker genes are still required to match cell types constructed by the algorithm with actual biological cell types. Complete deconvlution also lowers the cost compared to methods that require cell-type proportions as input, which is typically estimated by using costly antibodies and flow cytometry.

In addition, our probabilistic model is conceptually more advanced than methods using matrix decomposition [15–17] or regression techniques [1, 25] for a couple reasons. First, our generative model explicitly considers how reads are generated and estimates cell proportions instead of RNA proportions whereas matrix decomposition or regression-based methods are not. Second, our model employs multinomial random variables to capture the stochastic nature of reads and therefore inherently builds in the constraint that proportions are nonnegative and sum to one on the parameters of interest; whereas matrix decomposition or regression-based methods need to impose those constraints on the parameter space explicitly, which brings technical challenges for numerical procedures.

Our proposed model extended the original LDA model in two primary ways that would be unnecessary in the context of natural language processing, but are crucial for RNA-Seq data. First, we built in a dependence of gene expression on gene length. Second, we accommodated possibly different amounts of RNA per cell from cell types whose cells differ in size when estimating the proportion of cells of each type in the sample. In addition, instead of specifying the number of cell types a priori, we provided an algorithm that allows the data to guide selection of the number of cell types. Finally, we proposed a quasi-unsupervised learning strategy that augments the input data (GEPs from mixed samples) with additional GEPs from pure cell lines that are anticipated to be components of the mixture.

We systematically compared the performance of CDSeq with seven competing deconvolution methods: CIBERSORT [25], DeconRNAseq [22], deconf [16], ssKL [17], UNDO [29], DSA [20] and csSAM [1]. Our comparisons encompassed a range of data sets: synthetic mixtures created numerically from GEPs of pure cell lines, GEPs measured on heterogeneous RNA samples constructed in our lab by mixing RNA extracted from pure cell lines in different proportions, the experimental expression data that was used to evaluate csSAM, expression data of 22 leukocyte subtypes (LM22) [25], expression data from follicular lymphoma samples [25], and expression data from samples of peripheral blood mononuclear cells (PBMC) [25]. In all these comparisons, CDSeq performed as well or better than competitors in estimating of cell-type proportions and cell-type-specific GEPs from heterogeneous tissue samples.

CDSeq, an unsupervised data mining tool, is fully data-driven and allows simultaneous estimation of both cell-type-specific GEPs and sample-specific cell mixing proportions. In some real data analyses when constituent cell types had highly correlated GEPs, the cell types found by CDSeq lacked a one-to-one correspondence with the known component cell lines. Our quasi-unsupervised approach ameliorates this problem. It involves augmenting the available GEPs from heterogeneous samples with GEPs from pure cultures of the cell types anticipated to be constituents. We showed that this quasi-unsupervised approach can improve CDSeq’s performance in lymphoma and deep deconvolution examples. In practice, whether or not to apply quasi-unsupervised approach would depend on the goal of the study. If a user is interested in deep deconvolution where one would like to know the proportions of related cell subtypes (e.g., different T subpopulations in samples), then the quasi-unsupervised approach would be recommended. In this case, the appended pure cell line GEPs should be those of the T cell subpopulations. Furthermore, inclusion of such cell line GEPs does not exclude identification of cell types other than those appended pure cell lines.

To improve CDSeq’s computational efficiency, we developed a data dilution strategy that can speed up the algorithm while retaining the accuracy of estimation (S1 Methods and S9 Fig). Furthermore, filtering out genes with low expression levels or with little sample-to-sample variation will reduce the running time and memory usage. CDSeq often manages to finish within couple hours. Currently, CDSeq is coded in MATLAB and Octave. An R package is currently being developed for a broader accessibility.

A limitation of current CDSeq model is the impossibility of fine tuning the hyperparameters to obtain optimal results without ground truth. In practice, we suggest setting *α* = 5, *β* = 0.5. When heterogeneous samples are likely dominated by one or two cell types, setting *α* < 1 may help; when cell-type-specific GEPs are likely to have relatively high correlation, setting *β* > 1 may help—though we cannot specify a definitive threshold for high correlation. From a practical point of view, the higher the correlations are, the fuzzier the discovered signal would be. Another potentially helpful technique is the quasi-unsupervised strategy. Efforts at enabling CDSeq to self-adjust hyperparameters based on given data are underway. Another possible extension for the current model is that the fundamental multinomial model used for gene expression imposes a certain negative correlation between expression counts at different loci. However, it is conceivable that, because genetic pathways can be regulated as units, the counts could be positively correlated among certain subsets of genes. The current CDSeq model cannot handle that kind of correlation structure.

In addition, the RNA-Seq mixtures generated in this work can serve as a valuable benchmarking dataset for other deconvolution methods.

We expect that CDSeq will prove valuable for analysis of cellular heterogeneity on bulk RNA-Seq data. This computational method provides a practical and promising alternative to methods that require expensive laboratory apparatus and extensive labor to isolate individual cells from heterogeneous samples, which could also entail possible loss of a systems perspective. Application of CDSeq will aid in deciphering complex genomic data from heterogeneous tissues.

## Supporting information

S1 Methods Statistical inference for CDSeq. (PDF)

S1 Table Randomly generated sample-specific cell-type proportions (%) used to create synthetic data. (PDF)

S2 Table Cell-type (RNA) proportions (%) used to create mixed samples in the experiment with cultured cell types. (PDF)

S1 Fig Results for synthetic data. (PDF)

S2 Fig Results for experimental data. (PDF)

S3 Fig Results for liver, brain and lung mixtures data. (PDF)

S4 Fig Deconvolution of 22 leukocyte subtypes (LM22) data set. (PDF)

S5 Fig Correlation among true LM22 GEPs. (PDF)

S6 Fig Result of deconvolution of follicular lymphoma tumors data. (PDF)

S7 Fig Results of deconvolution of 14 follicular lymphoma tumors samples. (PDF)

S8 Fig Performance comparisons on deep deconvolution. (PDF)

S9 Fig Running time of CDSeq plotted against the dilution factor for data dilution using the synthetic data and experimental data. (PDF)

S10 Fig Linearity assumption test using 32 experimental mixtures. (PDF)
