# Supplementary material for: CDSeq: A novel complete deconvolution method for dissecting heterogeneous samples using gene expression data
Source: PLoS Comput Biol. 2019 Dec 2;15(12):e1007510. doi: 10.1371/journal.pcbi.1007510 (PMC6907860; doi:10.1371/journal.pcbi.1007510)
Supplement: S1 Methods — (PDF) [file pcbi.1007510.s001.pdf]

Supporting information for CDSeq: A novel complete deconvolution method for dissecting heterogeneous samples using gene expression data

Kai Kang[1], Qian Meng[1], Igor Shats[2], David M. Umbach[1], Melissa Li[1], Yuanyuan Li[1], Xiaoling Li[2], Leping Li[1]

[1]*Biostatistics and Computational Biology Branch, NIH/NIEHS*

[2]*Signal Transduction Lab, NIH/NIEHS*

Supplementary Methods

Notations	
M	Number of tissue samples
G	Total number of genes
N_i	Total number of reads for sample i
$\bar{N}_t$	Total number of reads assigned to cell type t for all samples
θ_i	Multinomial parameters that describe the sample-specific proportions of the cell types for sample i, $\theta_i \in \mathbb{R}_+^T$, $\sum_{t=1}^{T} \theta_{i,t} = 1$and let $\theta = (\theta_{i,t})_{i=1,t=1}^{M,T}$
ϕ_t	Multinomial parameters that describe the cell-type-specific gene expression for cell type t, $\phi_t \in \mathbb{R}_+^G$, $\sum_{e=1}^{G} \phi_{t,e} = 1$ and let $\phi = (\phi_{t,e})_{t=1,e=1}^{T,G}$
$\alpha = (\alpha_i)_{i=1}^{M}$	Dirichlet parameter, hyperparameter for θ_i
$\beta = (\beta_e)_{e=1}^{G}$	Dirichlet parameter, hyperparameter for ϕ_t
$r_{i,j}$	mapped RNA-seq read j for sample i, let $r = (r_{i,j})_{i=1,j=1}^{M,N_i}$
$c_{i,j}$	cell type assignment for read $r_{i,j}$, $c_{i,j} \in \{1, \cdots, T\}$ and let $c = (c_{i,j})_{i=1,j=1}^{M,N_i}$
η_t	Poisson parameter that describes the amount of RNA generated from cell type t, let $\eta = (\eta_t)_{t=1}^{T}$
ℓ_e	gene length for gene e, let $\ell = (\ell_e)_{e=1}^{G}$
$\tilde{\ell}_e$	effective gene length for gene e, $\tilde{\ell}_e = \ell_e - m + 1$, where m is the fixed sequencing length, let $\tilde{\ell} = (\tilde{\ell}_e)_{e=1}^{G}$
$g_{i,j}$	mapped gene of read $r_{i,j}$, $g_{i,j} \in \{1, \cdots, G\}$ and let $g = (g_{i,j})_{i=1,j=1}^{M,N_i}$

Notations (continue)	
$gid(r_a)$	mapping from a read r_a to its gene. We sometimes dropped the double subscripts i, j for convenience and use $r = \{r_1, \cdots, r_n\}$ to denote all the reads from all samples
$cid(r_a)$	mapping from a read r_a to its cell type assignment
$sid(r_a)$	mapping from a read r_a to its sample

Parameter estimation Based on our model, given the hyperparameters, reads alignment, effective lengths of the mapped genes and the estimate of η, the joint distribution of the parameters of interest is

$$\begin{aligned} & p(\phi, \theta, c, r, g|\alpha, \beta, \eta, \tilde{\ell}) \\ = & \prod_{t=1}^{T} p(\phi_t|\beta) \prod_{i=1}^{M} \left(p(\theta_i|\alpha) \prod_{j=1}^{N_i} p(c_{i,j}|\theta_i, \eta) p(r_{i,j}, g_{i,j}|\phi, \tilde{\ell}, c_{i,j}) \right) \\ = & \prod_{t=1}^{T} p(\phi_t|\beta) \prod_{i=1}^{M} \prod_{j=1}^{N_i} p(r_{i,j}, g_{i,j}|\phi, \tilde{\ell}, c_{i,j}) \prod_{i=1}^{M} \left(p(\theta_i|\alpha) \prod_{j=1}^{N_i} p(c_{i,j}|\theta_i, \eta) \right). \end{aligned}$$

Integrating out θ and ϕ, we have

$$\begin{aligned} & p(c, r, g|\alpha, \beta, \eta, \tilde{\ell}) \\ = & \underbrace{\int_{\phi} \prod_{t=1}^{T} p(\phi_t|\beta) \prod_{i=1}^{M} \prod_{j=1}^{N_i} p(r_{i,j}, g_{i,j}|\phi, \tilde{\ell}, c_{i,j}) d\phi}_{p(r,g|c,\beta,\tilde{\ell})} \underbrace{\int_{\theta} \prod_{i=1}^{M} \left(p(\theta_i|\alpha) \prod_{j=1}^{N_i} p(c_{i,j}|\theta_i, \eta) \right) d\theta}_{p(c|\alpha,\eta)}. \end{aligned}$$

Subsequently,

$$p(r, g|c, \beta, \tilde{\ell})$$

$$= \int_{\phi} \prod_{t=1}^{T} p(\phi_t|\beta) \prod_{i=1}^{M} \prod_{j=1}^{N_i} p(r_{i,j}, g_{i,j}|\phi, \tilde{\ell}, c_{i,j}) d\phi \quad (1)$$

$$= \prod_{t=1}^{T} \int_{\phi_t} \frac{\Gamma(\sum_{e=1}^{G} \beta_e)}{\prod_{e=1}^{G} \Gamma(\beta_e)} \left(\prod_{e=1}^{G} \phi_{t,e}^{\beta_e - 1} \right) \left(\prod_{\tau=1}^{\bar{N}_t} \frac{\phi_{t,g_{i_\tau,j_\tau}} \tilde{\ell}_{g_{i_\tau,j_\tau}}}{\sum_{e=1}^{G} \phi_{t,e} \tilde{\ell}_e} \frac{1}{\tilde{\ell}_{g_{i_\tau,j_\tau}}} \right) d\phi_t, \quad (2)$$

where $\bar{N}_t$ denotes the number of reads assigned to cell type t, and i_τ, j_τ denote the sample id and read index within the sample i_τ respectively, g_{i_τ,j_τ} and $\tilde{\ell}_{g_{i_\tau,j_\tau}}$ denote the gene assignment and its effective length from which the read r_{i_τ,j_τ} is generated. From (1) to (2), it is done by grouping the reads according to their cell type assignments and writing the Dirichlet distribution for ϕ_t explicitly. Similarly, we have that

$$p(c|\alpha, \eta)$$

$$= \int_{\theta} \prod_{i=1}^{M} \left(p(\theta_i|\alpha) \prod_{j=1}^{N_i} p(c_{i,j}|\theta_i, \eta) \right) d\theta$$

$$= \prod_{i=1}^{M} \int_{\theta_i} \frac{\Gamma(\sum_{t=1}^{T} \alpha_t)}{\prod_{t=1}^{T} \Gamma(\alpha_t)} \left(\prod_{t=1}^{T} \theta_{i,t}^{\alpha_t - 1} \right) \prod_{j=1}^{N_i} \prod_{t=1}^{T} \left(\frac{\theta_{i,t} \eta_t}{\sum_{t=1}^{T} \theta_{i,t} \eta_t} \right)^{\mathbb{1}\{c_{i,j}=t\}} d\theta_i, \quad (3)$$

where $\mathbb{1}\{\cdot\}$ is the indicator function takes values of 1 or 0. Ideally, one could integrate out the parameters θ and ϕ separately. Nevertheless, such direct computation is complicated by the fact that $p(r_{i,j}, g_{i,j}|\phi, \tilde{\ell}, c_{i,j})$ and $p(c_{i,j}|\theta_i, \eta)$ contain normalizing denominators as shown in (2) and (3).

To simplify the computation, we define $\hat{\phi}_{t,g_{i_\tau,j_\tau}} = \frac{\phi_{t,g_{i_\tau,j_\tau}} \tilde{\ell}_{g_{i_\tau,j_\tau}}}{\sum_{k=1}^{G} \phi_{t,k} \tilde{\ell}_k}$ and $\hat{\theta}_{i,t} = \frac{\theta_{i,t}\eta_t}{\sum_{t=1}^{T} \theta_{i,t}\eta_t}$. Notice that $\hat{\phi}_t$ and $\hat{\theta}_i$ are random variables on simplex. We therefore use two Dirichlet random variables $\tilde{\phi}_t = (\tilde{\phi}_{t,1}, \cdots, \tilde{\phi}_{t,G})$ and $\tilde{\theta}_i = (\tilde{\theta}_{i,1}, \cdots, \tilde{\theta}_{i,T})$, to approximate $\hat{\phi}_t$ and $\hat{\theta}_i$. We assume they are characterized by known hyperparameters $\tilde{\beta}, \tilde{\alpha}$. Then, we can first perform the statistical inference

on the surrogate parameters $\tilde{\phi}, \tilde{\theta}$. In details,

$$
\begin{aligned}
& p(r, g|c, \tilde{\beta}, \tilde{\ell}) \\
= & \prod_{t=1}^{T} \int_{\tilde{\phi}_t} p(\tilde{\phi}_t|\tilde{\beta}) \prod_{\tau=1}^{\bar{N}_t} p(r_{i_\tau j_\tau}, g_{i_\tau j_\tau}|\tilde{\phi}_t, \tilde{\ell}, c_{i_\tau j_\tau} = t) d\tilde{\phi}_t \\
= & \prod_{t=1}^{T} \int_{\phi_t} \frac{\Gamma(\sum_{i=1}^{G} \tilde{\beta}_i)}{\prod_{i=1}^{G} \Gamma(\tilde{\beta}_i)} \left(\prod_{i=1}^{G} \tilde{\phi}_{t,i}^{\tilde{\beta}_i - 1} \right) \left(\prod_{\tau=1}^{\bar{N}_t} \tilde{\phi}_{t, g_{i_\tau, j_\tau}} \right) d\tilde{\phi}_t \\
= & \prod_{t=1}^{T} \int_{\phi_t} \frac{\Gamma(\sum_{e=1}^{G} \tilde{\beta}_e)}{\prod_{e=1}^{G} \Gamma(\tilde{\beta}_e)} \left(\prod_{e=1}^{G} \tilde{\phi}_{t,e}^{\tilde{\beta}_e - 1} \right) \left(\prod_{\tau=1}^{\bar{N}_t} \prod_{e=1}^{G} \tilde{\phi}_{t,e}^{\mathbb{1}\{g_{i_\tau, j_\tau} = e\}} \right) d\tilde{\phi}_t \\
= & \prod_{t=1}^{T} \frac{\Gamma(\sum_{e=1}^{G} \tilde{\beta}_e)}{\prod_{e=1}^{G} \Gamma(\tilde{\beta}_e)} \frac{\prod_{e=1}^{G} \Gamma(\tilde{\beta}_e + \sum_{\tau=1}^{\bar{N}_t} \mathbb{1}\{g_{i_\tau, j_\tau} = e\})}{\Gamma(\sum_{e=1}^{G} \tilde{\beta}_e + \sum_{e=1}^{G} \sum_{\tau=1}^{\bar{N}_t} \mathbb{1}\{g_{i_\tau, j_\tau} = e\})},
\end{aligned} \tag{4}
$$

similarly, we have

$$
p(c|\tilde{\alpha}, \eta) = \prod_{i=1}^{M} \frac{\Gamma(\sum_{t=1}^{T} \tilde{\alpha}_t)}{\prod_{t=1}^{T} \Gamma(\tilde{\alpha}_t)} \frac{\prod_{t=1}^{T} \Gamma(\tilde{\alpha}_t + \sum_{j=1}^{N_i} \mathbb{1}\{c_{i,j} = t\})}{\Gamma(\sum_{t=1}^{T} \tilde{\alpha}_t + \sum_{t=1}^{T} \sum_{j=1}^{N_i} \mathbb{1}\{c_{i,j} = t\})}. \tag{5}
$$

We employed a Gibbs sampler to draw samples from the posterior distribution on the cell type assignments of all the reads from all samples. Therefore, we need the conditional distribution $p(c_a|c_{-a}, r, g)$, where r denotes the reads of all samples and c_{-a} denotes the cell type assignments without assigning the ath read. In particular, assume the total number of reads is n, then $c = (c_1, \cdots, c_n)$ denotes the cell type assignment for all reads $r = (r_1, \cdots, r_n)$ from all the samples. To be clear, we use $gid(r_a), sid(r_a)$ as references to the gene id and sample id of read r_a respectively, and $cid(r_a)$ refers to the corresponding cell type assignment used in the calculation

when needed. Apply eqn.(4) and eq.(5) and the fact $\Gamma(x+1) = x\Gamma(x)$, we have

$$
\begin{aligned}
& p(c_a | c_1, \cdots, c_{a-1}, c_{a+1}, \cdots, c_n, r, g) \\
= & \frac{p(c_1, \cdots, c_n, r, g)}{p(c_1, \cdots, c_{a-1}, c_{a+1}, c_n, r, g)} \\
\propto & \frac{\beta_{gid(r_a)} + \sum\limits_{\tau=1}^{N_{c_a}} \mathbb{1}_{-1}\{g_{i_\tau, j_\tau} = gid(r_a)\}}{\sum\limits_{e=1}^{G} \beta_e + \sum\limits_{e=1}^{G} \sum\limits_{\tau=1}^{N_{c_a}} \mathbb{1}_{-1}\{g_{i_\tau, j_\tau} = e\}} \frac{\alpha_{c_a} + \sum\limits_{j=1}^{N_{sid(r_a)}} \mathbb{1}_{-1}\{c_{i,j} = c_a\}}{\sum\limits_{t=1}^{T} \alpha_t + \sum\limits_{t=1}^{T} \sum\limits_{j=1}^{N_{sid(r_a)}} \mathbb{1}_{-1}\{c_{i,j} = t\}}
\end{aligned}
\tag{6}
$$

where $\sum\limits_{\tau=1}^{N_{c_a}} \mathbb{1}_{-1}\{g_{i_\tau, j_\tau} = gid(r_a)\}$ denotes the counts of all the reads with gene id $gid(r_a)$ that assigned to cell type c_a without the single count of current assignment of the read r_a itself. Finally, we can estimate the parameters based on the posterior predictive distribution for new data as follows,

$$
\begin{aligned}
\tilde{\phi}_{t,e} &= \frac{\beta_e + \sum\limits_{\tau=1}^{\bar{N}_t} \mathbb{1}\{g_{i_\tau, j_\tau} = e\}}{\sum\limits_{k=1}^{G} \beta_k + \sum\limits_{k=1}^{G} \sum\limits_{\tau=1}^{\bar{N}_t} \mathbb{1}\{g_{i_\tau, j_\tau} = k\}}, \\
\tilde{\theta}_{i,t} &= \frac{\alpha_t + \sum\limits_{j=1}^{N_i} \mathbb{1}\{c_{i,j} = t\}}{\sum\limits_{k=1}^{T} \alpha_k + \sum\limits_{k=1}^{T} \sum\limits_{j=1}^{N_i} \mathbb{1}\{c_{i,j} = k\}},
\end{aligned}
$$

where $t = 1, \cdots, T$ denotes cell type, $e = 1, \cdots, G$ denotes gene id, and $i = 1, \cdots, M$ denote sample id, and $\sum\limits_{\tau=1}^{\bar{N}_t} \mathbb{1}\{gid(r_{i_\tau, j_\tau}) = e\}$ is the count of reads from gene e that are assigned to cell type t, and $\sum\limits_{j=1}^{N_i} \mathbb{1}\{c_{i,j} = t\}$ is the count of reads from sample i that are assigned to cell type t. Then we can recover the desired parameter ϕ, θ

$$
\begin{aligned}
\phi_{t,e} &= \frac{\tilde{\phi}_{t,e} / \tilde{\ell}_e}{\sum_{k=1}^{G} \tilde{\phi}_{t,k} / \tilde{\ell}_k}, \\
\theta_{i,t} &= \frac{\tilde{\theta}_{i,t} / \eta_t}{\sum_{k=1}^{T} \tilde{\theta}_{i,k} / \eta_k}.
\end{aligned}
$$

To infer the CDSeq-identified cell types, reference gene expression profiles of pure cell lines (raw read count preferred for RPKM normalization) is required. One can use correlation as a metric to associate the cell type with the cell types in the reference profile.

Determine the number of cell types using the data The number of cell types present in a heterogenous sample may not be known in advance. Our model allows the number of cell types to be inferred from the data. We formulate this inference as a problem of model selection [1, 2]. Using our statistical model, assuming the hyperparameters α, β and the reads mapping information are known. Let $\mathcal{C}$ denote the space of all possible cell type assignments for all the reads, then summing over $c \in \mathcal{C}$, the joint probability distribution of the cell type-specific gene expression profiles, the sample-specific proportions of the cell types, and the mapped reads is given in eqn. (7).

$$
\begin{aligned}
& p(r, g, \theta, \phi | \alpha, \beta, \eta, \tilde{\ell}) \\
= \; & \sum_{c \in \mathcal{C}} p(r, g, c, \theta, \phi | \alpha, \beta, \eta, \tilde{\ell}) \\
= \; & \sum_{c \in \mathcal{C}} \prod_{t=1}^{T} p(\phi_t | \beta) \prod_{i=1}^{M} \left(p(\theta_i | \alpha) \prod_{j=1}^{N_i} p(c_{i,j} | \theta_i, \eta) p(r_{i,j}, g_{i,j} | \phi, \tilde{\ell}, c_{i,j}) \right) \\
= \; & \prod_{t=1}^{T} p(\phi_t | \beta) \prod_{i=1}^{M} \left(p(\theta_i | \alpha) \prod_{j=1}^{N_i} \sum_{c \in \mathcal{C}} p(c_{i,j} | \theta_i, \eta) p(r_{i,j}, g_{i,j} | \phi, \tilde{\ell}, c_{i,j}) \right) \\
= \; & \prod_{t=1}^{T} p(\phi_t | \beta) \prod_{i=1}^{M} \left(p(\theta_i | \alpha) \prod_{j=1}^{N_i} \sum_{c_{i,j}=1}^{T} p(c_{i,j} | \theta_i, \eta) p(r_{i,j}, g_{i,j} | \phi, \tilde{\ell}, c_{i,j}) \right) \\
= \; & \prod_{t=1}^{T} p(\phi_t | \beta) \prod_{i=1}^{M} \left(p(\theta_i | \alpha) \prod_{j=1}^{N_i} \sum_{c_{i,j}=1}^{T} \frac{\theta_{i,c_{i,j}} \eta_{c_{i,j}}}{\sum\limits_{t=1}^{T} \theta_{i,t} \eta_t} \frac{\phi_{c_{i,j}, g_{i,j}} \tilde{\ell}_{g_{i,j}}}{\sum\limits_{k=1}^{G} \phi_{t,k} \tilde{\ell}_k} \right)
\end{aligned}
$$

$$= \prod_{t=1}^{T} p(\phi_t|\beta) \prod_{i=1}^{M} \left(p(\theta_i|\alpha) \prod_{e=1}^{G} \left(\sum_{t=1}^{T} \frac{\theta_{i,t}\eta_t}{\sum_{\tau=1}^{T} \theta_{i,\tau}\eta_\tau} \frac{\phi_{t,e}\tilde{\ell}_e}{\sum_{k=1}^{G} \phi_{t,k}\tilde{\ell}_k} \right)^{n_{i,e}} \right)$$

$$= \prod_{t=1}^{T} \frac{\Gamma(\sum_{e=1}^{G} \beta_e)}{\prod_{e=1}^{G} \Gamma(\beta_e)} \left(\prod_{e=1}^{G} \phi_{t,e}^{\beta_e - 1} \right) \prod_{i=1}^{M} \left(\frac{\Gamma(\sum_{t=1}^{T} \alpha_t)}{\prod_{t=1}^{T} \Gamma(\alpha_t)} \left(\prod_{t=1}^{T} \theta_{i,t}^{\alpha_t - 1} \right) \prod_{e=1}^{G} \left(\sum_{t=1}^{T} \frac{\theta_{i,t}\eta_t}{\sum_{\tau=1}^{T} \theta_{i,\tau}\eta_\tau} \frac{\phi_{t,e}\tilde{\ell}_e}{\sum_{k=1}^{G} \phi_{t,k}\tilde{\ell}_k} \right)^{n_{i,e}} \right)$$

$$\propto \prod_{t=1}^{T} \left(\prod_{e=1}^{G} \phi_{t,e}^{\beta_e - 1} \right) \prod_{i=1}^{M} \left(\left(\prod_{t=1}^{T} \theta_{i,t}^{\alpha_t - 1} \right) \prod_{e=1}^{G} \left(\sum_{t=1}^{T} \frac{\theta_{i,t}\eta_t}{\sum_{\tau=1}^{T} \theta_{i,\tau}\eta_\tau} \frac{\phi_{t,e}\tilde{\ell}_e}{\sum_{k=1}^{G} \phi_{t,k}\tilde{\ell}_k} \right)^{n_{i,e}} \right), \quad (7)$$

where $n_{i,e}$ denotes the count of the reads mapped to gene e in sample i. Then taking logarithmic of the posterior in (7), we have

$$\log p(r, \theta, \phi|\alpha, \beta, \eta, \ell, g)$$

$$= \sum_{t=1}^{T} \sum_{e=1}^{G} (\beta_e - 1) \log(\phi_{t,e}) + \sum_{i=1}^{M} \left(\sum_{t=1}^{T} (\alpha_t - 1) \log \theta_{i,t} + \sum_{e=1}^{G} n_{i,e} \log \left(\sum_{t=1}^{T} \frac{\theta_{i,t}\eta_t}{\sum_{\tau=1}^{T} \theta_{i\tau}\eta_\tau} \frac{\phi_{t,e}\tilde{\ell}_e}{\sum_{k=1}^{G} \phi_{t,k}\tilde{\ell}_k} \right) \right)$$

$$= \underbrace{\sum_{t=1}^{T} \sum_{e=1}^{G} (\beta_e - 1) \log(\phi_{t,e}) + \sum_{i=1}^{M} \sum_{t=1}^{T} (\alpha_t - 1) \log \theta_{i,t}}_{\text{regulation terms imposed by prior distributions}} + \underbrace{\sum_{i=1}^{M} \sum_{e=1}^{G} n_{i,e} \log \left(\sum_{t=1}^{T} \frac{\theta_{i,t}\eta_t}{\sum_{k=1}^{T} \theta_{i,k}\eta_k} \frac{\phi_{t,e}\tilde{\ell}_e}{\sum_{k=1}^{G} \phi_{t,k}\tilde{\ell}_k} \right)}_{\text{likelihood of the data}}. \quad (8)$$

The log posterior of RNA-seq data given in (8) can be simplified as follows

$$h(\tilde{\theta}, \tilde{\phi}, r)$$

$$= \sum_{t=1}^{T} \sum_{e=1}^{G} (\tilde{\beta}_e - 1) \log(\tilde{\phi}_{t,e}) + \sum_{i=1}^{M} \sum_{t=1}^{T} (\tilde{\alpha}_t - 1) \log \tilde{\theta}_{i,t} + \sum_{i=1}^{M} \sum_{e=1}^{G} n_{i,e} \log \left(\sum_{t=1}^{T} \tilde{\theta}_{i,t}\tilde{\phi}_{t,e} \right), \quad (9)$$

Given a set of candidate values for the number of cell types present in the mixture samples, CDSeq will choose the one that maximizes eqn (9).

In general, Bayesian priors (the regulation term in eqn.(8)) can be understood as a way to lessen the chance of overfitting. The regulation term (or penalty term) reflects the competition between prior belief and the observed data. The more data we have, the less effect of the prior belief would be on the estimations. In practice, it is reasonable to assume that the number of cell types are between 20 and 30. This number would be smaller if one is only interested in the major cell types. From our experience, the log posterior had only one maximum and exhibited bell-like shape trajectory in general.

Cell type association The output of CDSeq reports cell types that are anonymous – in the sense of not being identified with actual cell types. To match the CDSeq-identified cell types to actual cell type, a list of reference cell-type-specific GEPs and metric of similarity (for example, Pearson's correlation coefficient or Kullback-Leibler divergence) is required. We employed Pearson's correlation coefficient as the similarity measurement. Ideally, each estimated cell type will have a high correlation (say exceeding 0.6) with exactly one reference cell type so that the matching is straightforward. In practice, the CDSeq-identified cell types cannot always be uniquely assigned to actual cell types. In such cases, the Munkres algorithm [3] can be employed to yield one-to-one cell type associations (Munkres algorithm was included in current version of CDSeq). In some cases, one-to-one cell type association is not immediate (S9 fig and S10 Fig), because a CDSeq-identified cell type may highly correlated with multiple actual cell types. Then, the sample-specific

proportion for an actual cell type can be estimated by combining all the proportions of the CDSeq-identified cell types that are highly correlated (say greater than 0.6 or other user defined threshold) with that cell type. We adopted root mean square error (RMSE) as the performance assessment measurement which is defined as

$$\text{RMSE}(\hat{x}, x) = \sqrt{\frac{\sum_{i=1}^{n}(\hat{x}_i - x_i)^2}{n}} \tag{10}$$

where $\hat{x}$ denotes the estimated parameter, x denotes the true parameter and n denotes the dimension of the parameter. In all the comparisons, we computed the RMSE of estimated GEPs in the original RPKM scale instead of $log2$ scale.

A data dilution strategy to speed up the algorithm Often data sets of RNA-seq raw counts are large; the total reads across all samples could range from millions to billions. Since CDSeq's Gibbs sampler is running on the space of all the raw reads, excessively large data sets could dramatically slow the algorithm or even kill it if memory requirements exceed capacity. To address this issue, we propose a data dilution strategy: we divide all the read counts by a positive constant and round them to integer values (since CDSeq requires positive integers as input). We showed that our dilution strategy can speed up the algorithm while retaining the accuracy of estimation.

We systematically increased the dilution factor from 100 to 5000 with an increment of 50 for the synthetic data and from 10 to 100 with an increment of 1 for the experimental dataset. We have a couple reasons for using smaller dilution factors for the experimental dataset. First, the sequencing depth of our pure cell lines is about 10 times higher than that of the experimental dataset. Second, the synthetic mixtures were generated in silico with no technical noise. Taken

together, a large dilution factor for sequencing data with low depth would jeopardize estimation accuracy. For the synthetic dataset, we demonstrated that the estimated cell-type-specific gene expression profiles (csGEPs) and sample-specific proportions (SSP) remain reasonably accurate even when the dilution factor is as high as 500. The correlations between CDSeq-estimates and true csGEPs and SSP are greater than 0.94. This is also true for the experimental data when the dilution factor is smaller than 20. The correlations between CDSeq-estimates and true csGEPs and SSP are higher than 0.88 (S9 Fig).

Synthetic mixtures We generated synthetic gene expression profiles for 40 synthetic mix-ture samples using gene expression profiles for six pure cell lines. We downloaded expression profiles from the CSHL (Cold Spring Harbor Laboratory) website for: normal fetal lung fibroblast, normal B-lymphocyte, normal mammary epithelial cells, normal umbilical vein endothelial cells, breast epithelial carcinoma, and normal CD14-positive cells from human leukapheresis production. To artificially amplify the confounding factor of differences in cell-type-specific RNA quantity, we multiplied the cell-line reads counts by a predefined vector to rescale the RNA amounts. Specifically, we randomly chose to double RNA-seq reads count of the normal B lymphocyte and normal mammary epithelial breast cell lines. We then randomly generated mixing proportions (S1 Table) that specified the propor-tion of cells of each type in each synthetic sample using a Dirichlet distribution with a parameter vector having all six entries equal to 5. The six pure cell lines' GEPs are available upon request or can be found download information on https://github.com/kkang7/CDSeq.

Experimental mixtures and gene expression profiling MCF7 cells were obtained from Duke University Cell culture facility and cultured in DMEM medium supplemented with 10% fetal bovine serum (FBS). Namalwa cells were a gift from Dr. Sandeep Dave, Duke University, and were cultured in RPMI medium supplemented with 10% FBS. Hs343T and hTERT-HME1 (ATCC) were cultured in HuMEC Ready medium (Thermo Fisher Scientific).

In brief, total mRNA was prepared from Namalwa (Burkitt's lymphoma), Hs343T (fibroblast line derived from a mammary gland adenocarcinoma), hTERT-HME1 (normal mammary epithelial cells immortalized with hTERT), and MCF7 (estrogen receptor positive breast cancer cell line). mRNA samples were diluted to 100 $ng/\mu\ell$ and mixed in different proportions (S2 Table). Global mRNA abundance of the four pure cell lines and of the mixed RNA samples was profiled by RNA-sequencing.

Sequencing libraries were prepared using TruSeq RNA sample preparation kit v2 (Illumina). 75-bp single end sequencing was performed on the NextSeq sequencer (Illumina). After obtaining the fastq data, we first ran cutadapt [4] (version 1.12) for trimming adapter sequences. Secondly, we mapped reads to the genome using STAR [5] (version 020201). Lastly, we used featureCounts [6] (version 1.5.1) to generate raw read counts data as the input for our algorithm. The code for processing the fastq data using cutadapt, STAR and featureCounts are available at https://github.com/kkang7/CDSeq.

Based on RNA-sequencing, we found contamination in the pure cell lines by examining the gene expression of KRT5. KRT5, a marker specific to HME-hTERT, should be completely absent

from cancer-associated fibroblasts (CAFs); however, it was present in CAF samples at about 20% of the levels found in hTERT-HME1. This contamination is possibly attributable to CAFs being derived from tumors so that they were probably contaminated with a small portion of tumor tissue. In vitro, this small portion of tumor cells had huge growth advantage and, thus, became significant. In summary, CAF samples were not pure but contained about 20% RNA from HME-like cells. We are not certain if the contamination comes from hTERT-HME1 cells or other cancer cells which express endogenous TERT. To alleviate this problem, we considered the proportions of CAF (given in S2 Table) to be 80% of CAF and 20% of HME and adjusted the proportions accordingly in the comparisons (Fig 4 in main text, S2 Fig and S3 Fig). Datasets are available at the GEO repository (GSE123604).

Deconvolution methods for comparison We compared our results to those of csSAM [7], CIBERSORT [8], DSA [9], DeconRNAseq [10], UNDO [11], deconf [12], and ssKL [13] when applicable. We downloaded the csSAM R package and used the default settings for all simulations and comparisons. The proportion information provided to csSAM was either known in advance or estimated using flow cytometry. On the other hand, CIBERSORT, based on the same linearity argument, applies a support vector regression method, called ν-support vector regression [14]. It takes as input the gene expression profile of the bulk tissue samples and a gene expression profile for each possible cell type that comprises the bulk tissue; it outputs an estimate of the cell-type proportions for each sample. In addition, to study the fractions of immune cells, a gene expression signature profile for 22 cell types, named LM22, was proposed by CIBERSORT. We requested the source code for CIBERSORT from the authors and ran all comparisons with default settings. When

GEPs of pure cell lines that constitute the heterogeneous samples were available, we provided them to CIBERSORT. DSA employed quadratic programming for GEPs estimation by requiring either marker genes of the cell types in the mixtures or cell type proportions of those cell types in samples. We installed DSA R package and used default settings for all examples. We provided the sample-specific cell-type proportions to DSA when available. DeconRNAseq takes bulk gene expression and cell-type-specific GEPs as input and estimates sample-specific cell-type proportions using quadratic programming. We installed DeconRNAseq R package and used default settings. We provided true GEPs to DeconRNAseq in all simulations and comparisons. UNDO estimates tumor-stroma proportions using linear latent variable model. We applied the UNDO R package using default settings in all cases when applicable. deconf and ssKL estimate both sample-specific cell-type proportions and cell-type-specific GEPs using nonnegative matrix factorization. We installed deconf R package using its source code and applied default settings in all studies.

Most deconvolution methods assume the following linearity in their models, i.e. $X_{ij} = \sum_{t=1}^{T} w_{it}h_{tj} + e_{ij}$, where X_{ij} is the expression for bulk sample i and gene j and w_{it} denotes the proportion of cell type t for sample i and h_{tj} denotes the cell-type-specific gene expression for cell type t and gene j. Many methods, such as csSAM and CIBERSORT, used microarray data. In our work, for easy comparisons between different methods, we provided the RPKM normalization instead of raw read counts data to these methods. We validated the linearity assumption using our experimental dataset by comparing the RPKM of 32 mixtures to the RPKM of reconstituted mixtures using our four pure cell lines and known mixing proportions and plotted the results in S10 Fig. See [15] and references there in for discussions on data normalization for deconvolution.

References

[1] Griffiths, T. L. and Steyvers, M. (2004) Finding scientific topics. *Proceedings of the National academy of Sciences,* **101**(suppl 1), 5228–5235.

[2] Kass, R. E. and Raftery, A. E. (1995) Bayes factors. *Journal of the american statistical association,* **90**(430), 773–795.

[3] Burkard, R., Dell'Amico, M., and Martello, S. (2012) Assignment problems, revised reprint, Vol. 106, Siam, .

[4] Martin, M. (2011) Cutadapt removes adapter sequences from high-throughput sequencing reads. *EMBnet. journal,* **17**(1), pp–10.

[5] Dobin, A., Davis, C. A., Schlesinger, F., Drenkow, J., Zaleski, C., Jha, S., Batut, P., Chaisson, M., and Gingeras, T. R. (2013) STAR: ultrafast universal RNA-seq aligner. *Bioinformatics,* **29**(1), 15–21.

[6] Liao, Y., Smyth, G. K., and Shi, W. (2013) featureCounts: an efficient general purpose program for assigning sequence reads to genomic features. *Bioinformatics,* **30**(7), 923–930.

[7] Shen-Orr, S. S., Tibshirani, R., Khatri, P., Bodian, D. L., Staedtler, F., Perry, N. M., Hastie, T., Sarwal, M. M., Davis, M. M., and Butte, A. J. (2010) Cell type–specific gene expression differences in complex tissues. *Nature methods,* **7**(4), 287–289.

[8] Newman, A. M., Liu, C. L., Green, M. R., Gentles, A. J., Feng, W., Xu, Y., Hoang, C. D., Diehn, M., and Alizadeh, A. A. (2015) Robust enumeration of cell subsets from tissue expression profiles. *Nature methods,* **12**(5), 453.

[9] Zhong, Y., Wan, Y.-W., Pang, K., Chow, L. M., and Liu, Z. (2013) Digital sorting of complex tissues for cell type-specific gene expression profiles. *BMC bioinformatics,* **14**(1), 89.

[10] Gong, T. and Szustakowski, J. D. (2013) DeconRNASeq: a statistical framework for deconvolution of heterogeneous tissue samples based on mRNA-Seq data. *Bioinformatics,* **29**(8), 1083–1085.

[11] Wang, N., Gong, T., Clarke, R., Chen, L., Shih, I.-M., Zhang, Z., Levine, D. A., Xuan, J., and Wang, Y. (2014) UNDO: a Bioconductor R package for unsupervised deconvolution of mixed gene expressions in tumor samples. *Bioinformatics,* **31**(1), 137–139.

[12] Repsilber, D., Kern, S., Telaar, A., Walzl, G., Black, G. F., Selbig, J., Parida, S. K., Kaufmann, S. H., and Jacobsen, M. (2010) Biomarker discovery in heterogeneous tissue samples-taking the in-silico deconfounding approach. *BMC bioinformatics,* **11**(1), 27.

[13] Gaujoux, R. and Seoighe, C. (2012) Semi-supervised Nonnegative Matrix Factorization for gene expression deconvolution: a case study. *Infection, Genetics and Evolution,* **12**(5), 913–921.

[14] Schölkopf, B., Smola, A. J., Williamson, R. C., and Bartlett, P. L. (2000) New support vector algorithms. *Neural computation,* **12**(5), 1207–1245.

[15] Newman, A. M., Gentles, A. J., Liu, C. L., Diehn, M., and Alizadeh, A. A. (2017) Data normalization considerations for digital tumor dissection. *Genome biology,* **18**(1), 128.
