# Supplementary material for: CDSeq: A novel complete deconvolution method for dissecting heterogeneous samples using gene expression data
Source: PLoS Comput Biol. 2019 Dec 2;15(12):e1007510. doi: 10.1371/journal.pcbi.1007510 (PMC6907860; doi:10.1371/journal.pcbi.1007510)
Supplement: S1 Table — (PDF) [file pcbi.1007510.s002.pdf]

S1 Table. Randomly generated sample-specific cell-type proportions (%) used to create synthetic data.

	Cell types					
**Sample Number**	**Breast epithelial carcinoma**	**Normal B lymphocyte**	**Normal CD14+ leukapheresis**	**Normal fetal lung fibroblast**	**Normal mammary epithelial breast**	**Normal umbilical vein endothelial blood vessel**
1	16.1	16.0	10.3	18.0	15.1	24.4
2	16.3	22.2	20.2	15.0	10.7	15.7
3	16.8	24.4	16.2	20.2	5.4	16.9
4	14.5	26.2	15.8	14.0	15.1	14.3
5	20.5	18.8	19.3	8.0	13.9	19.5
6	20.1	21.7	8.7	14.9	29.5	5.1
7	14.8	21.3	21.7	21.9	8.6	11.7
8	15.6	8.1	28.9	16.1	14.7	16.6
9	11.4	8.4	12.7	24.8	15.8	26.9
10	12.8	25.4	18.8	15.0	9.8	18.3
11	15.7	19.9	11.4	14.7	19.1	19.3
12	9.2	26.2	9.7	18.1	20.4	16.4
13	8.3	19.1	20.5	18.4	6.8	26.9
14	21.3	26.6	11.1	18.9	9.7	12.5
15	31.5	16.8	14.0	15.3	12.2	10.2
16	22.0	18.4	11.4	9.8	19.7	18.8
17	12.4	18.3	11.8	26.5	14.8	16.2
18	8.5	18.3	16.1	22.0	16.7	18.3
19	6.6	8.2	10.8	10.4	44.7	19.3
20	21.3	29.1	5.2	22.3	12.6	9.5
21	17.7	14.7	25.2	17.5	12.6	12.2
22	17.5	33.3	11.2	8.2	20.1	9.7
23	9.0	13.9	30.2	23.4	9.4	14.1
24	13.1	17.7	26.5	17.6	12.6	12.6
25	18.6	12.8	14.5	10.4	16.5	27.1
26	20.1	14.4	16.7	12.2	21.1	15.6
27	28.0	21.5	11.6	14.7	16.3	8.0
28	16.9	13.7	16.5	22.4	5.8	24.8
29	35.4	16.2	11.5	12.2	19.0	5.7
30	18.9	20.9	11.9	15.4	11.7	21.1
31	10.2	10.2	22.7	18.2	19.7	19.1
32	8.4	13.6	11.1	19.8	22.4	24.7
33	13.7	6.7	19.1	13.1	23.9	23.5
34	15.7	7.5	22.2	24.7	15.1	14.9
35	23.0	20.0	13.9	10.8	8.3	24.0
36	10.1	34.1	14.3	25.9	9.7	5.9
37	20.7	24.3	11.4	15.2	14.3	14.2
38	14.6	30.2	19.2	15.6	9.0	11.4
39	17.3	24.9	14.3	14.2	8.5	20.8
40	9.4	9.9	15.4	31.4	19.4	14.5
