# Supplementary material for: CDSeq: A novel complete deconvolution method for dissecting heterogeneous samples using gene expression data
Source: PLoS Comput Biol. 2019 Dec 2;15(12):e1007510. doi: 10.1371/journal.pcbi.1007510 (PMC6907860; doi:10.1371/journal.pcbi.1007510)
Supplement: S2 Table — (PDF) [file pcbi.1007510.s003.pdf]

S2 Table. Cell-type (RNA) proportions (%) used to create mixed samples in the experiment with cultured cell types. Samples 1 to 4 and 37 to 40 are two replicates of the four pure cell lines. They were used as ground truth for benchmarking the CDSeq-identified cell types, not input for CDSeq

	**Cell types**			
**Sample Number**	**Tumor – MCF7**	**CAFs – Hs 343.T**	**Normal breast – hMECs-hTERT**	**Lymphocytes – Namalwa**
1	100	0	0	0
2	0	100	0	0
3	0	0	100	0
4	0	0	0	100
5	85	5	5	5
6	85	9	3	3
7	85	3	9	3
8	85	3	3	9
9	70	10	10	10
10	70	15	10	5
11	70	15	5	10
12	70	10	15	5
13	70	10	5	15
14	70	5	15	10
15	70	5	10	15
16	55	15	15	15
17	55	30	10	5
18	55	30	5	10
19	55	10	30	5
20	55	10	5	30
21	55	5	30	10
22	55	5	10	30
23	40	20	20	20
24	40	30	20	10
25	40	30	10	20
26	40	20	30	10
27	40	20	10	30
28	40	10	30	20
29	40	10	20	30
30	25	25	25	25
31	25	35	25	15
32	25	35	15	25
33	25	25	35	15
34	25	25	15	35
35	25	15	35	25
36	25	15	25	35
37	100	0	0	0
38	0	100	0	0
39	0	0	100	0
40	0	0	0	100
