# Supplementary material for: CDSeq: A novel complete deconvolution method for dissecting heterogeneous samples using gene expression data
Source: PLoS Comput Biol. 2019 Dec 2;15(12):e1007510. doi: 10.1371/journal.pcbi.1007510 (PMC6907860; doi:10.1371/journal.pcbi.1007510)
Supplement: S1 Fig — (PDF) [file pcbi.1007510.s004.pdf]

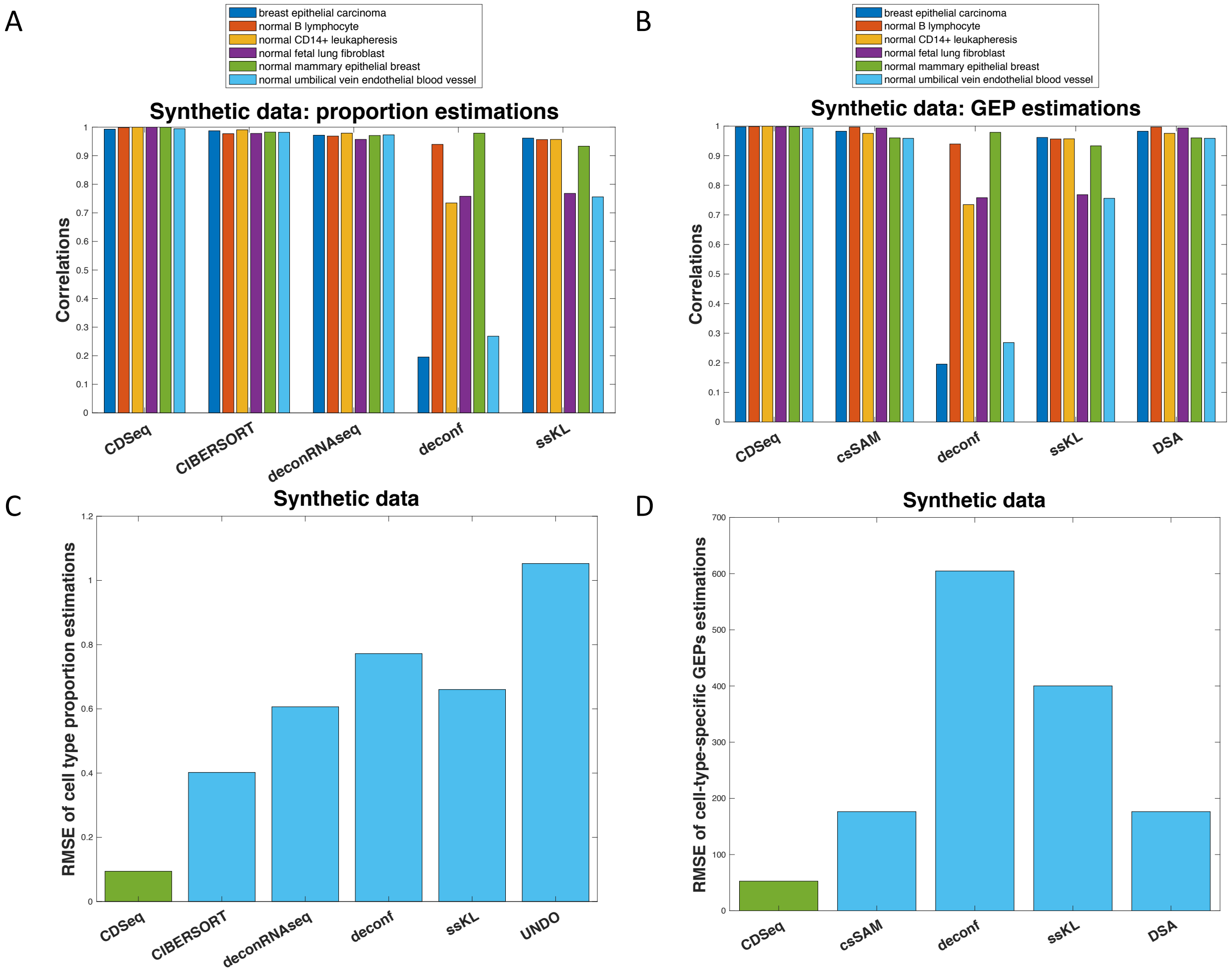

S1 Fig. Results for synthetic data. We ran CDSeq, CIBERSORT, csSAM, DeconRNAseq, UNDO, deconf, ssKL, and DSA on our synthetic mixtures. We set $\alpha = 5, \beta = 0.5, N = 700$ for CDSeq, and used default settings for all other methods. (A). Correlations between estimated sample-specific proportion (SSP) of cell types with true SSP; (B). Correlations between estimated cell-type-specific GEPs (csGEPs) with true csGEPs; (C). RMSEs of SSP estimations; (D). RMSE of csGEPs estimations. Notice that we do not show the result for UNDO in (A) and (B) because UNDO only estimates the proportions of tumor and non-tumor cell types.
