# Supplementary material for: CDSeq: A novel complete deconvolution method for dissecting heterogeneous samples using gene expression data
Source: PLoS Comput Biol. 2019 Dec 2;15(12):e1007510. doi: 10.1371/journal.pcbi.1007510 (PMC6907860; doi:10.1371/journal.pcbi.1007510)
Supplement: S2 Fig — (PDF) [file pcbi.1007510.s005.pdf]

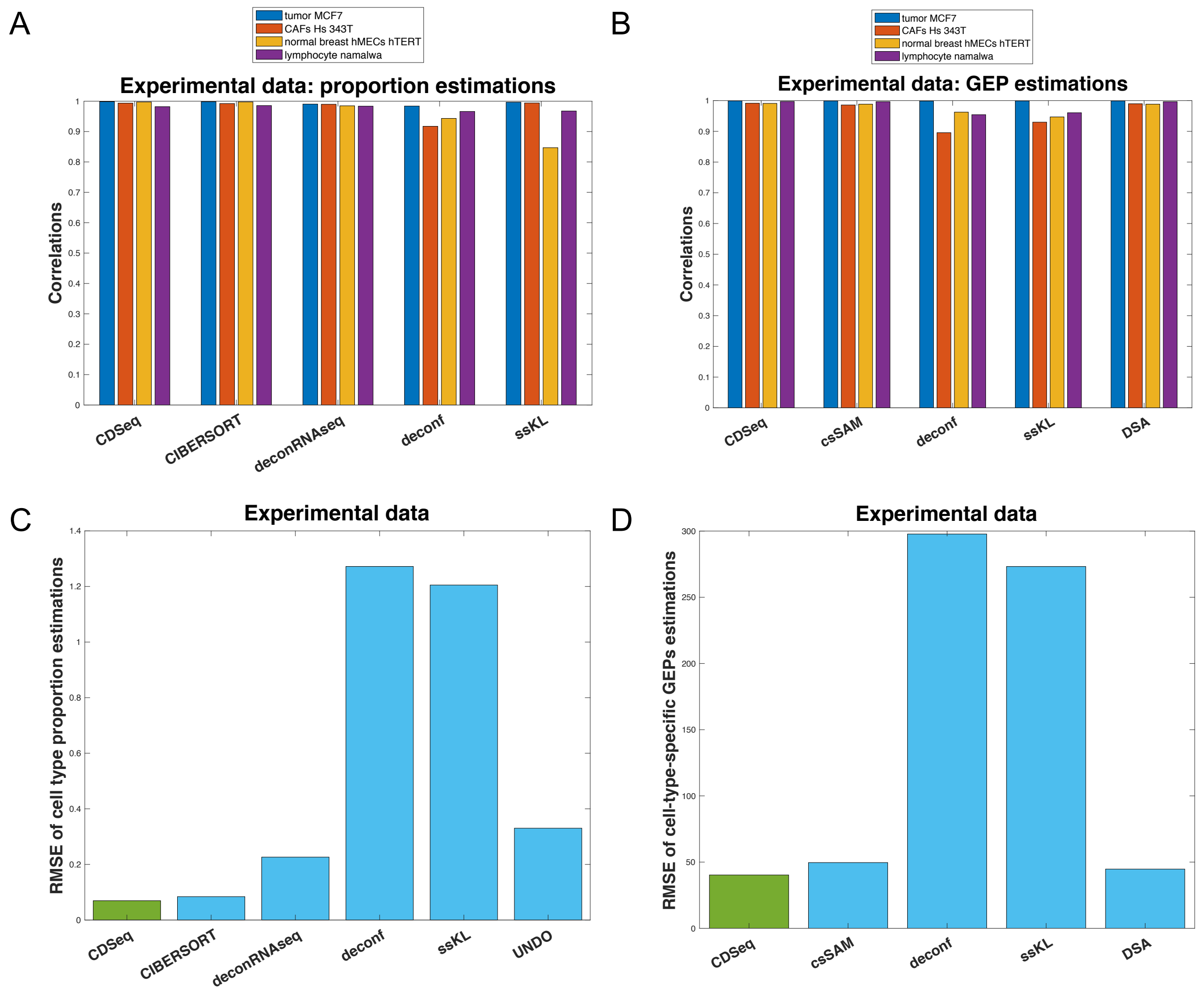

S2 Fig. Results for experimental data. We ran CDSeq, CIBERSORT, csSAM, DeconRNAseq, UNDO, deconf, ssKL, and DSA on our experimental mixtures. We set $\alpha = 5, \beta = 0.5, N = 700$ for CDSeq and used default settings for all other methods. (A). Correlations between estimated SSP with true SSP; (B). Correlations between estimated csGEPs with true csGEPs; (C). RMSEs of SSP estimations; (D). RMSEs of csGEPs estimation.
