# Supplementary material for: CDSeq: A novel complete deconvolution method for dissecting heterogeneous samples using gene expression data
Source: PLoS Comput Biol. 2019 Dec 2;15(12):e1007510. doi: 10.1371/journal.pcbi.1007510 (PMC6907860; doi:10.1371/journal.pcbi.1007510)
Supplement: S3 Fig — (PDF) [file pcbi.1007510.s006.pdf]

Dissecting mixtures of liver, lung and brain cells

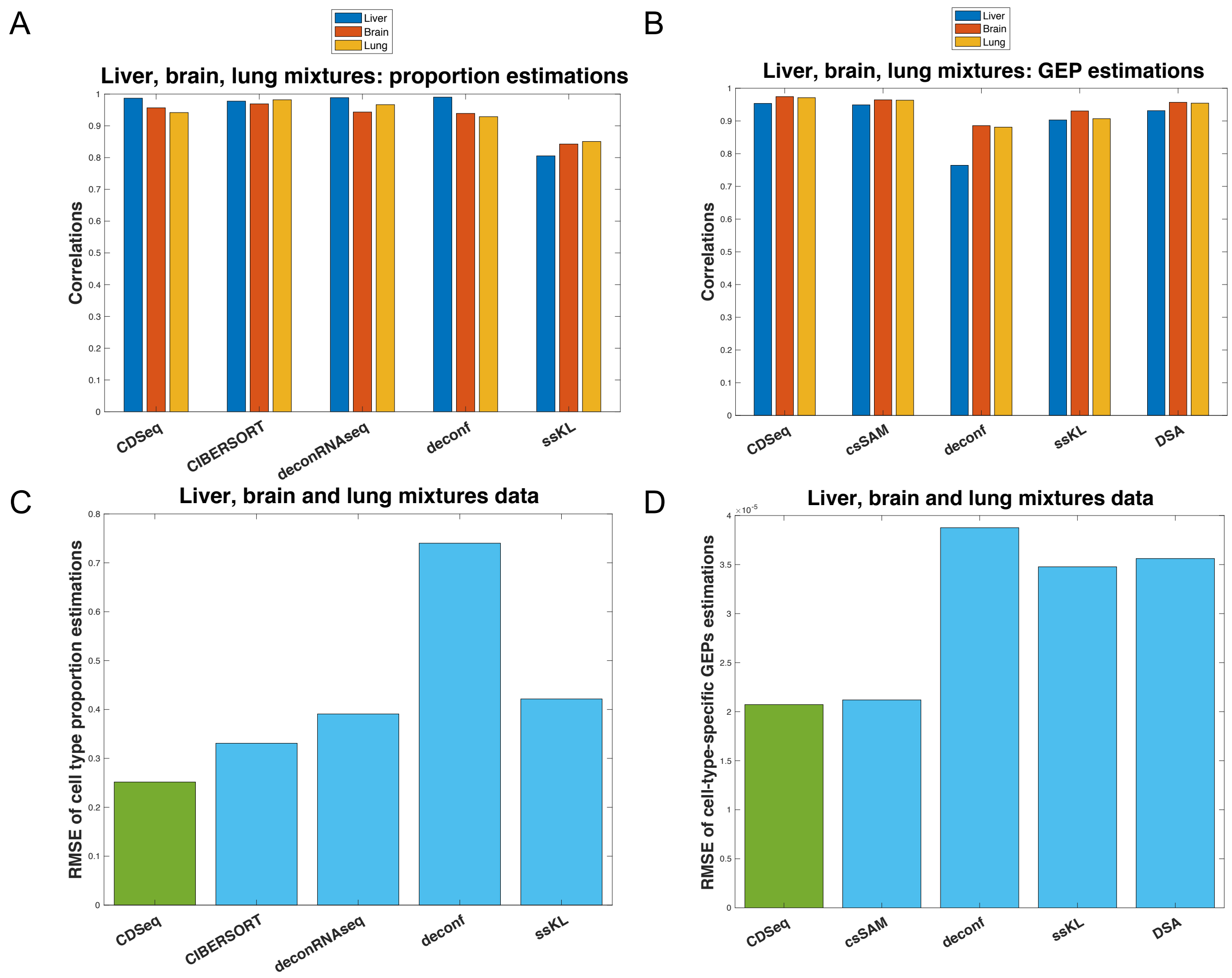

S3 Fig. Results for liver, brain and lung mixtures data. We ran CDSeq, CIBERSORT, csSAM, DeconRNAseq, UNDO, deconf, ssKL, and DSA on liver, brain, and lung mixtures. We set α=5, β=0.5, N=700 for CDSeq, and used default settings for all other methods. (A). Correlations between estimated SSP with true SSP; (B). Correlations between estimated csGEPs with true csGEPs; (C). RMSEs of SSP estimations; (D). RMSEs of csGEPs estimations
