# Supplementary material for: CDSeq: A novel complete deconvolution method for dissecting heterogeneous samples using gene expression data
Source: PLoS Comput Biol. 2019 Dec 2;15(12):e1007510. doi: 10.1371/journal.pcbi.1007510 (PMC6907860; doi:10.1371/journal.pcbi.1007510)
Supplement: S4 Fig — (PDF) [file pcbi.1007510.s007.pdf]

A

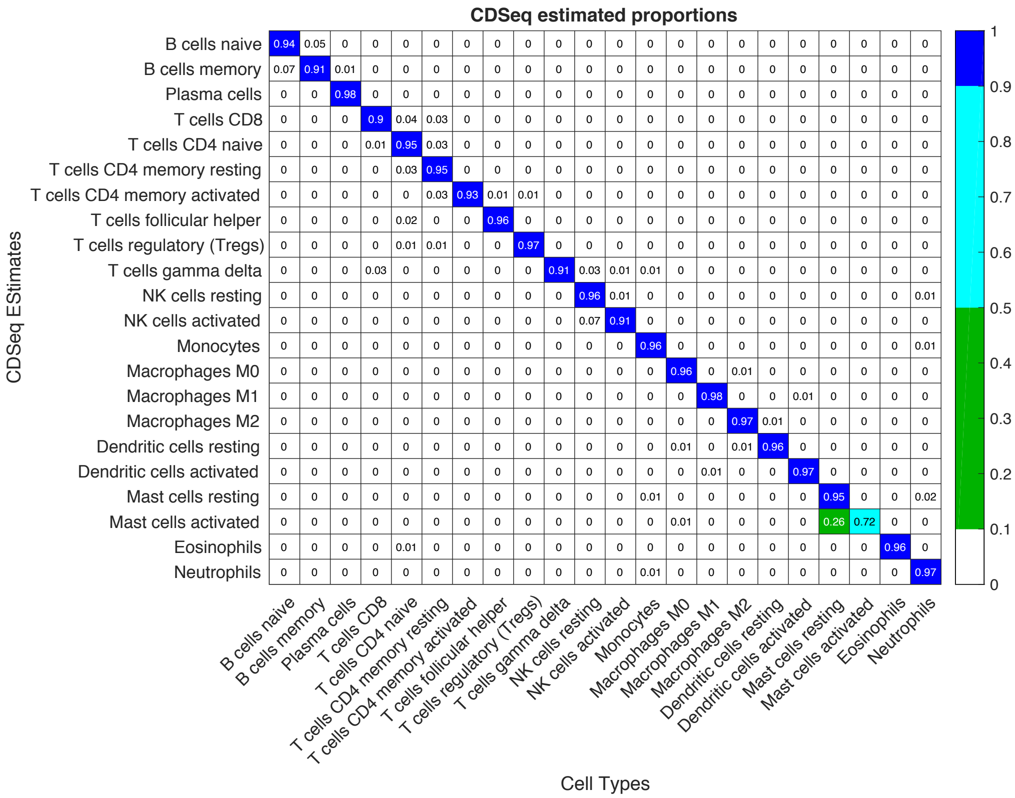
CDSeq estimated proportions
CDSeq EStimates
Cell Types
B cells naive
B cells memory
Plasma cells
T cells CD8
T cells CD4 naive
T cells CD4 memory resting
T cells CD4 memory activated
T cells follicular helper
T cells regulatory (Tregs)
T cells gamma delta
NK cells resting
NK cells activated
Monocytes
Macrophages M0
Macrophages M1
Macrophages M2
Dendritic cells resting
Dendritic cells activated
Mast cells resting
Mast cells activated
Eosinophils
Neutrophils
1
0.9
0.8
0.7
0.6
0.5
0.4
0.3
0.2
0.1
0

B

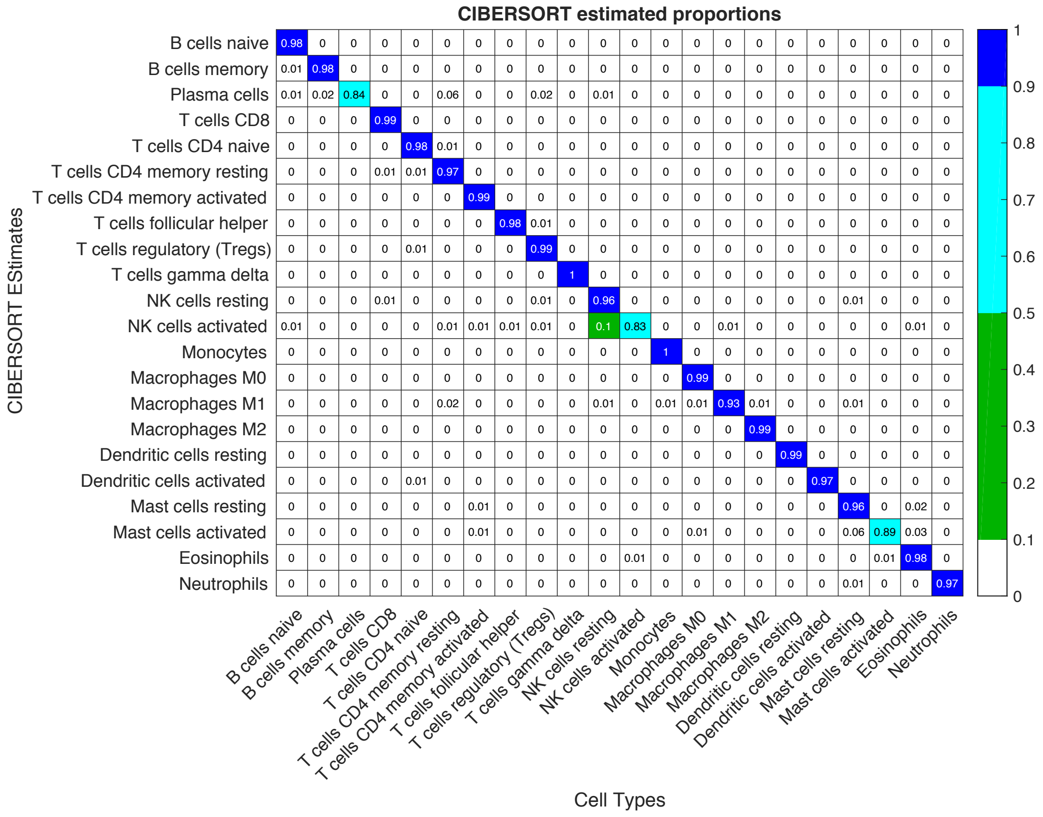
CIBERSORT estimated proportions
CIBERSORT EStimates
Cell Types
B cells naive
B cells memory
Plasma cells
T cells CD8
T cells CD4 naive
T cells CD4 memory resting
T cells CD4 memory activated
T cells follicular helper
T cells regulatory (Tregs)
T cells gamma delta
NK cells resting
NK cells activated
Monocytes
Macrophages M0
Macrophages M1
Macrophages M2
Dendritic cells resting
Dendritic cells activated
Mast cells resting
Mast cells activated
Eosinophils
Neutrophils
1
0.9
0.8
0.7
0.6
0.5
0.4
0.3
0.2
0.1
0

C

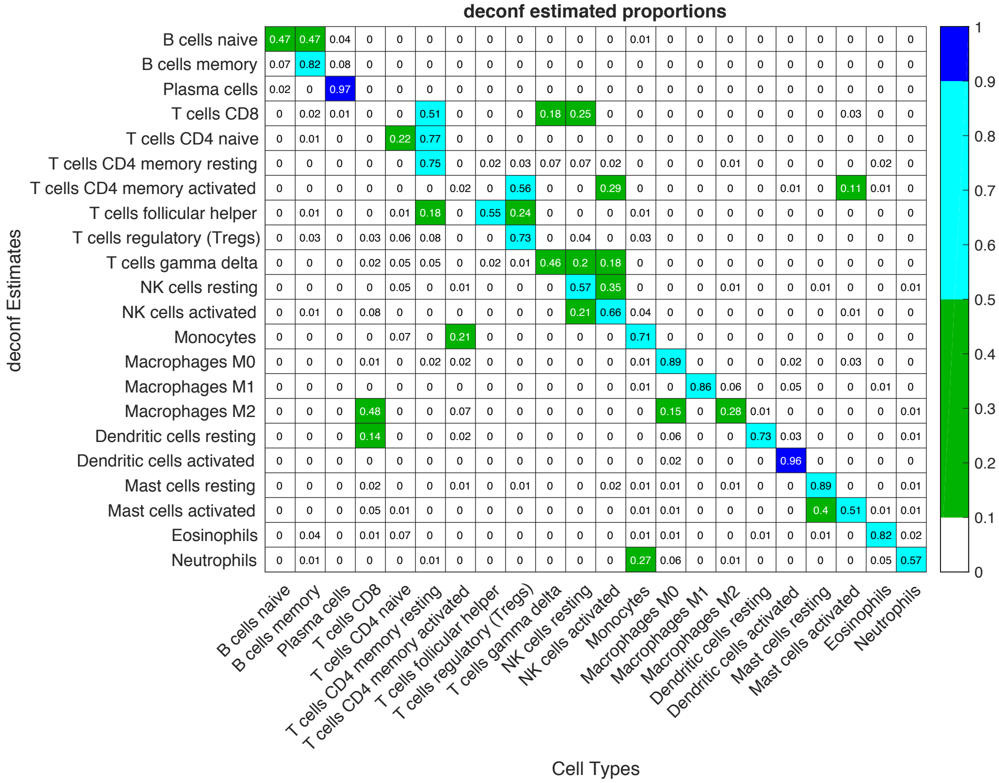
deconf estimated proportions
deconf Estimates
Cell Types
B cells naive
B cells memory
Plasma cells
T cells CD8
T cells CD4 naive
T cells CD4 memory resting
T cells CD4 memory activated
T cells follicular helper
T cells regulatory (Tregs)
T cells gamma delta
NK cells resting
NK cells activated
Monocytes
Macrophages M0
Macrophages M1
Macrophages M2
Dendritic cells resting
Dendritic cells activated
Mast cells resting
Mast cells activated
Eosinophils
Neutrophils

D

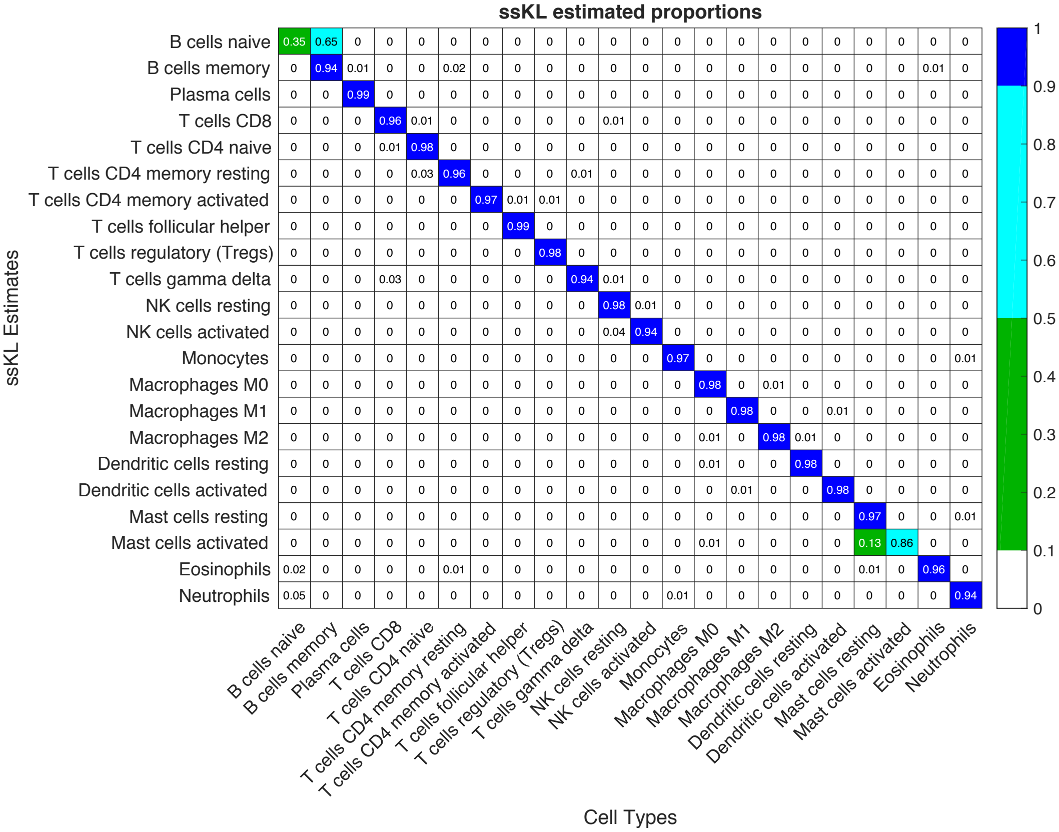
ssKL estimated proportions
ssKL Estimates
Cell Types
B cells naive
B cells memory
Plasma cells
T cells CD8
T cells CD4 naive
T cells CD4 memory resting
T cells CD4 memory activated
T cells follicular helper
T cells regulatory (Tregs)
T cells gamma delta
NK cells resting
NK cells activated
Monocytes
Macrophages M0
Macrophages M1
Macrophages M2
Dendritic cells resting
Dendritic cells activated
Mast cells resting
Mast cells activated
Eosinophils
Neutrophils

E

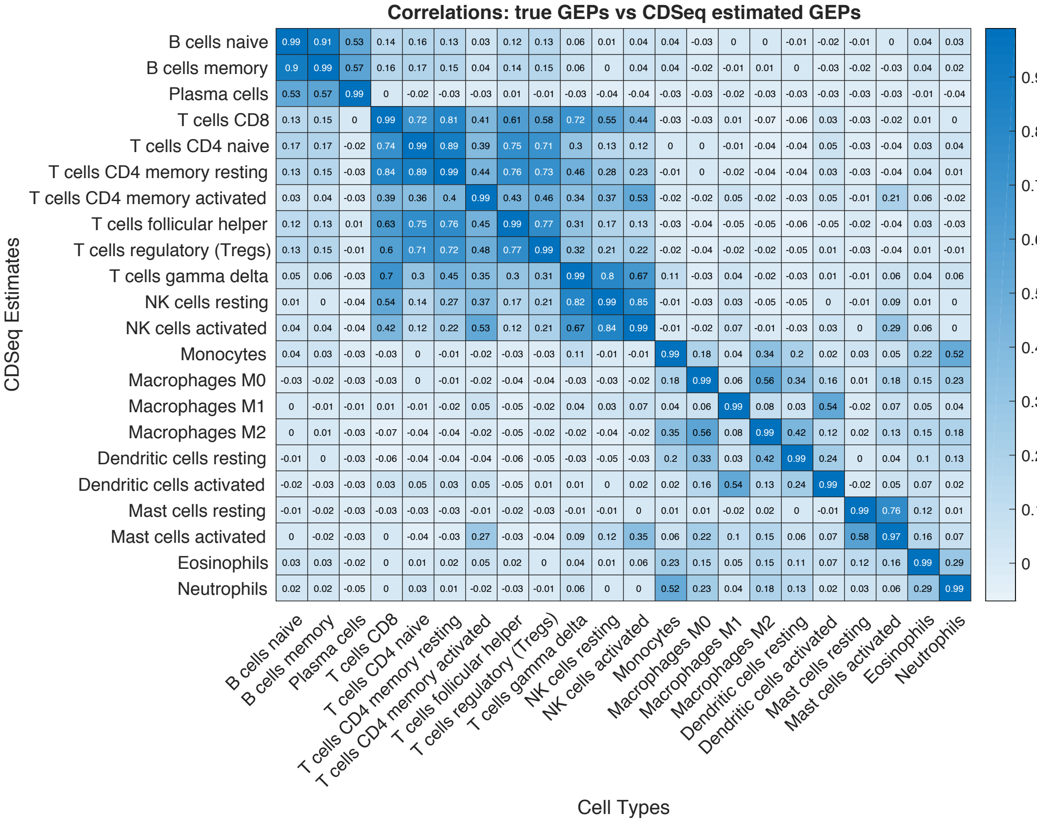
Correlations: true GEPs vs CDSeq estimated GEPs
CDSeq Estimates
Cell Types
B cells naive
B cells memory
Plasma cells
T cells CD8
T cells CD4 naive
T cells CD4 memory resting
T cells CD4 memory activated
T cells follicular helper
T cells regulatory (Tregs)
T cells gamma delta
NK cells resting
NK cells activated
Monocytes
Macrophages M0
Macrophages M1
Macrophages M2
Dendritic cells resting
Dendritic cells activated
Mast cells resting
Mast cells activated
Eosinophils
Neutrophils
0.9
0.8
0.7
0.6
0.5
0.4
0.3
0.2
0.1
0

F

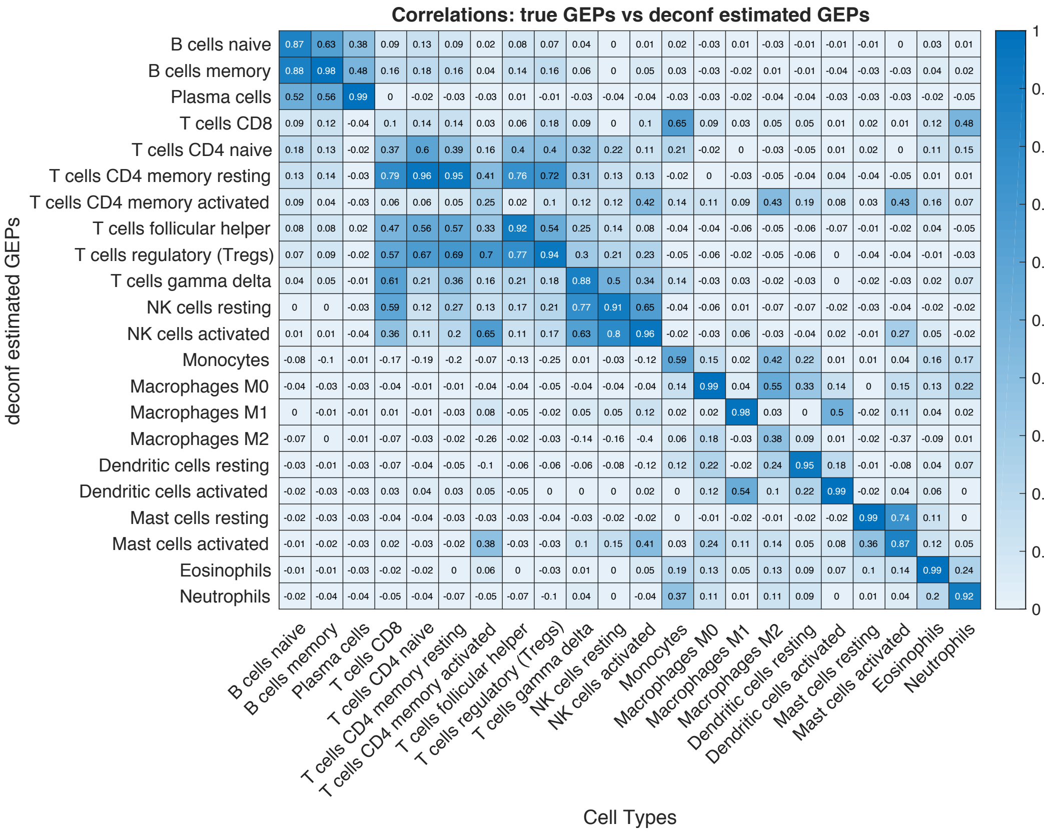
Correlations: true GEPs vs deconf estimated GEPs
deconf estimated GEPs
Cell Types
B cells naive
B cells memory
Plasma cells
T cells CD8
T cells CD4 naive
T cells CD4 memory resting
T cells CD4 memory activated
T cells follicular helper
T cells regulatory (Tregs)
T cells gamma delta
NK cells resting
NK cells activated
Monocytes
Macrophages M0
Macrophages M1
Macrophages M2
Dendritic cells resting
Dendritic cells activated
Mast cells resting
Mast cells activated
Eosinophils
Neutrophils
1
0.9
0.8
0.7
0.6
0.5
0.4
0.3
0.2
0.1
0

G

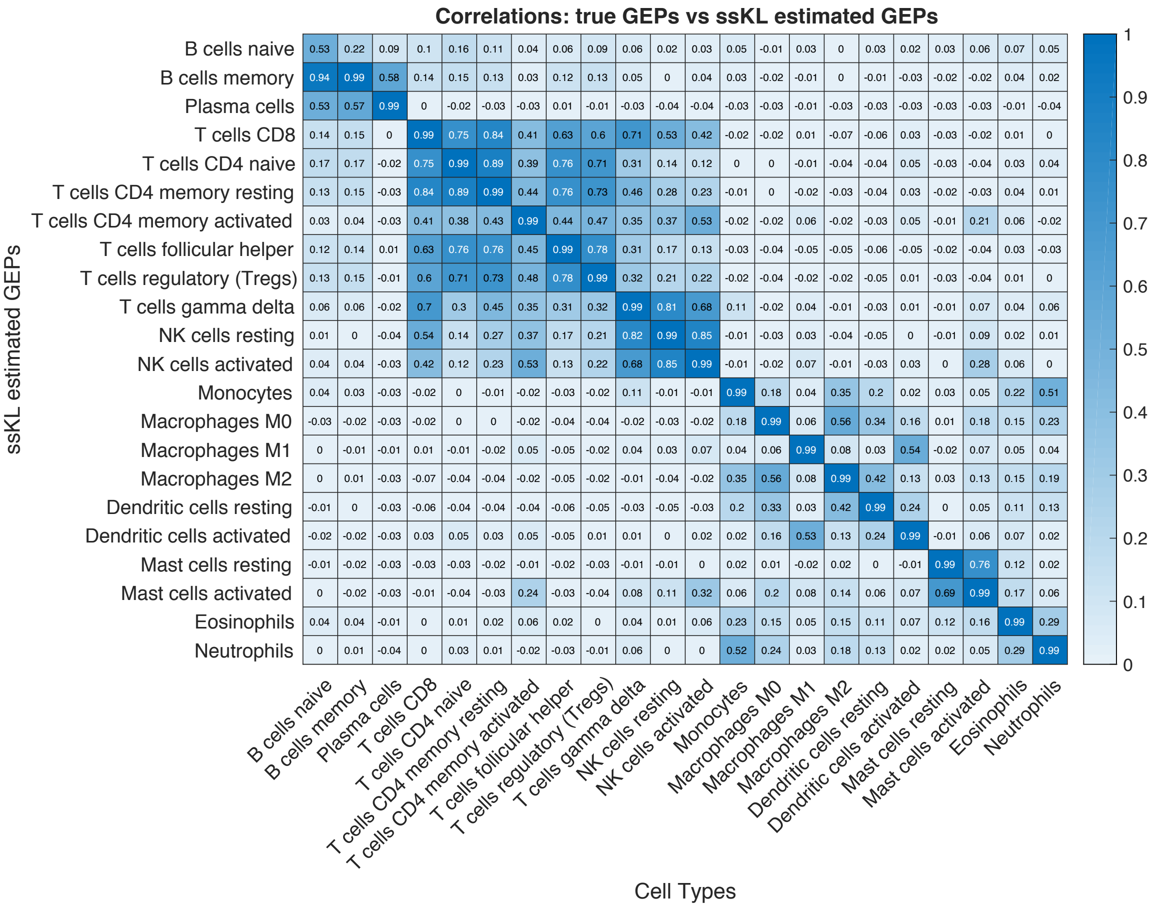


H

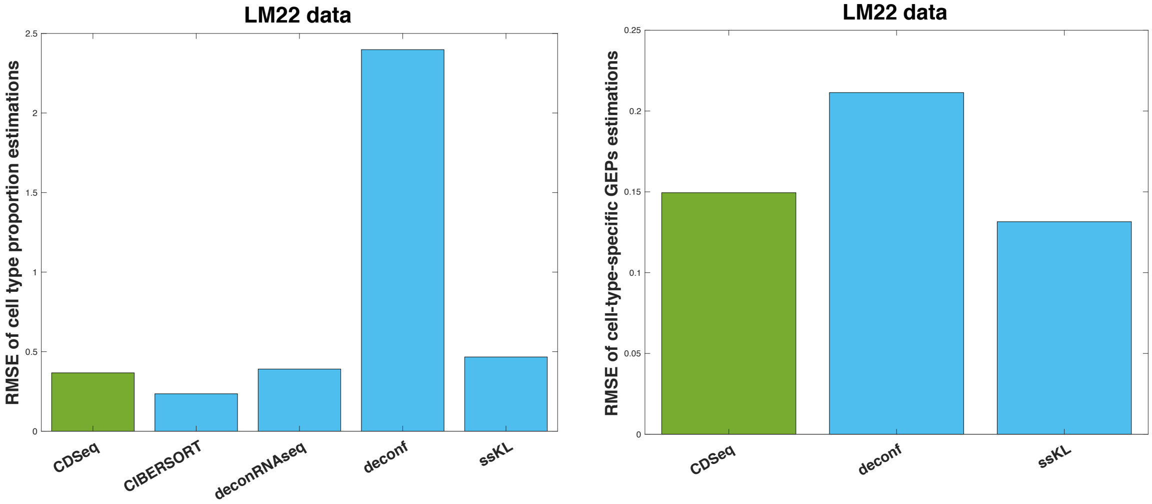


**S4 Fig. Deconvolution of 22 leukocyte subtypes (LM22) data set.** (A) Heatmap of CDSeq-estimated proportions of LM22 dataset. Each column represents a cell type in LM22 and the number at each row denotes the proportion of the corresponding cell type. The sums of proportions along the rows are not always equal to 1 owing to the fact that we rounded off all the proportions to 2 decimal places for better visual presentation. The proportions are largely, but not exclusively, dominated by a single cell type, indicating the accuracy of estimated proportions. (B) Heatmap of CIBERSORT-estimated proportions; (C) Heatmap of deconf-estimated proportions; (D) Heatmap of ssKL-estimated proportions; (E) Correlations of CDSeq-estimated GEPs versus true GEPs; (F) Correlations of deconf-estimated GEPs versus true

GEPs; (G) Correlations of ssKL-estimated GEPs versus true GEPs. (H) RMSEs of SSP estimations (left panel) and RMSEs of csGEPs estimations (right panel).
