# Supplementary figures and images for: CDSeq: A novel complete deconvolution method for dissecting heterogeneous samples using gene expression data
Source: PLoS Comput Biol. 2019 Dec 2;15(12):e1007510. doi: 10.1371/journal.pcbi.1007510 (PMC6907860; doi:10.1371/journal.pcbi.1007510)

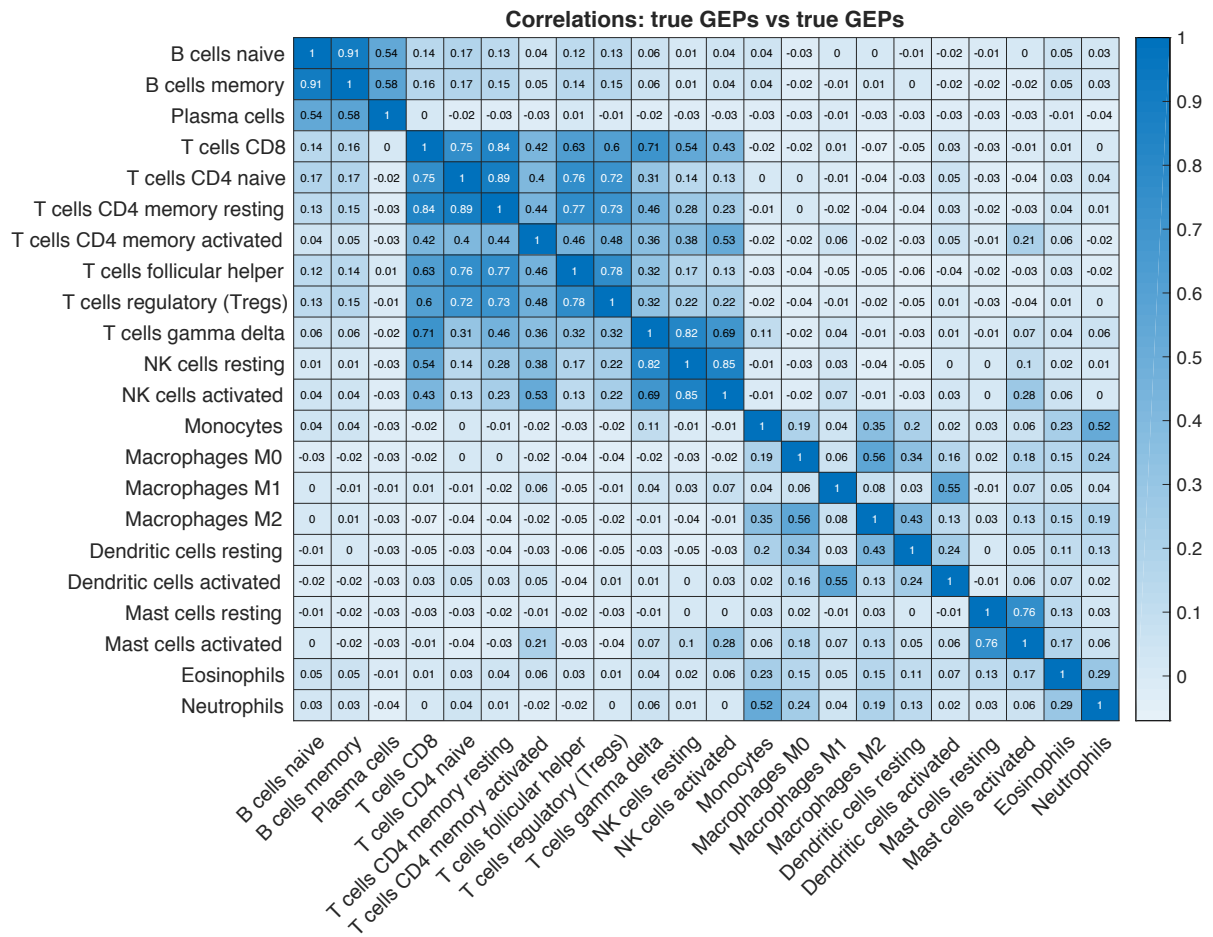

**S5 Fig. Correlation among true LM22 GEPs.** Some of the 22 cell types are highly correlated.

Supplement: S5 Fig — (PDF) [file pcbi.1007510.s008.pdf]
