# Supplementary material for: CDSeq: A novel complete deconvolution method for dissecting heterogeneous samples using gene expression data
Source: PLoS Comput Biol. 2019 Dec 2;15(12):e1007510. doi: 10.1371/journal.pcbi.1007510 (PMC6907860; doi:10.1371/journal.pcbi.1007510)
Supplement: S6 Fig — (PDF) [file pcbi.1007510.s009.pdf]

Immune cell analysis of lymphoma data with comparison to flow cytometry

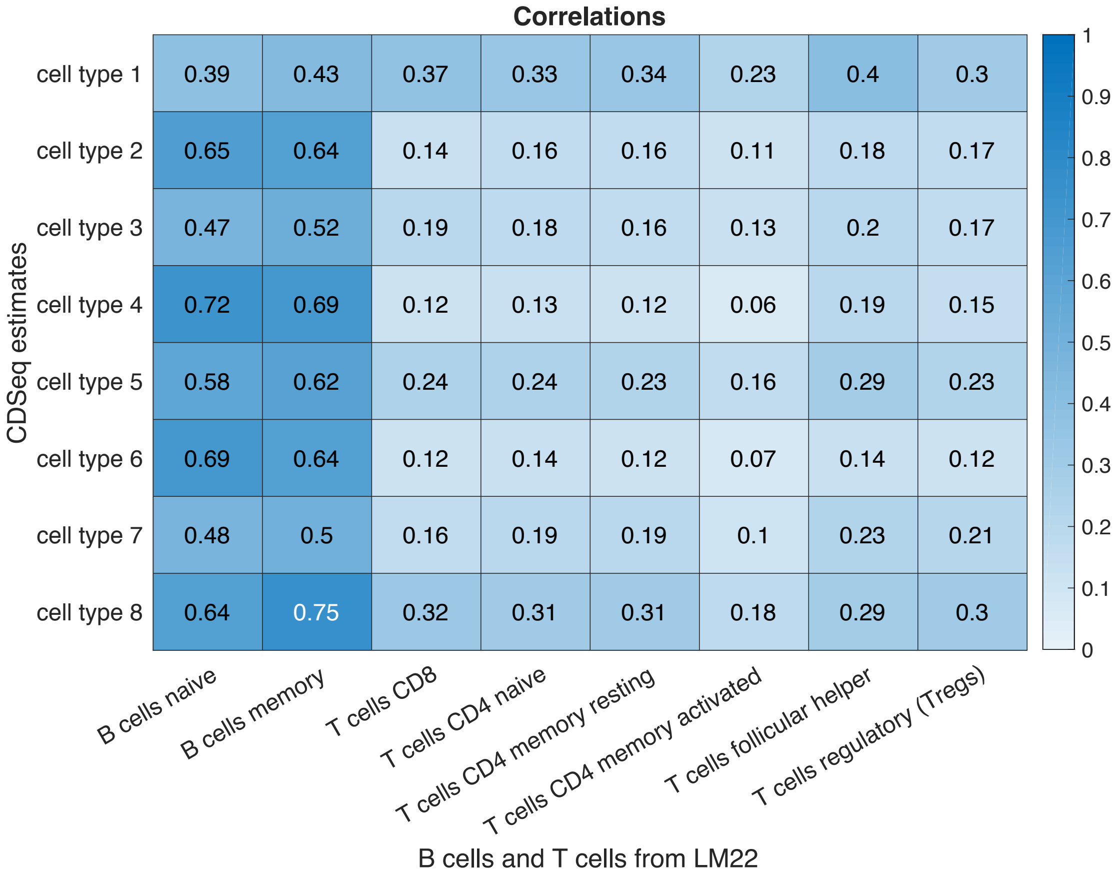

S6 Fig. Result of deconvolution of follicular lymphoma tumors data. Heatmap chart of correlations between CDSeq-estimated cell-type-specific GEPs using fully unsupervised mode. We set the number of cell types to be 8, hyperparameters $\alpha = 5, \beta = 0.5$, and 700 MCMC runs. The 8 CDSeq-identified cell-type-specific GEPs were relatively highly correlated with naïve B cell and memory B cell GEPs but less correlated with the T cell GEPs. In fully unsupervised mode, CDSeq could uncover signals only from B cells.
