# Supplementary material for: CDSeq: A novel complete deconvolution method for dissecting heterogeneous samples using gene expression data
Source: PLoS Comput Biol. 2019 Dec 2;15(12):e1007510. doi: 10.1371/journal.pcbi.1007510 (PMC6907860; doi:10.1371/journal.pcbi.1007510)
Supplement: S7 Fig — (PDF) [file pcbi.1007510.s010.pdf]

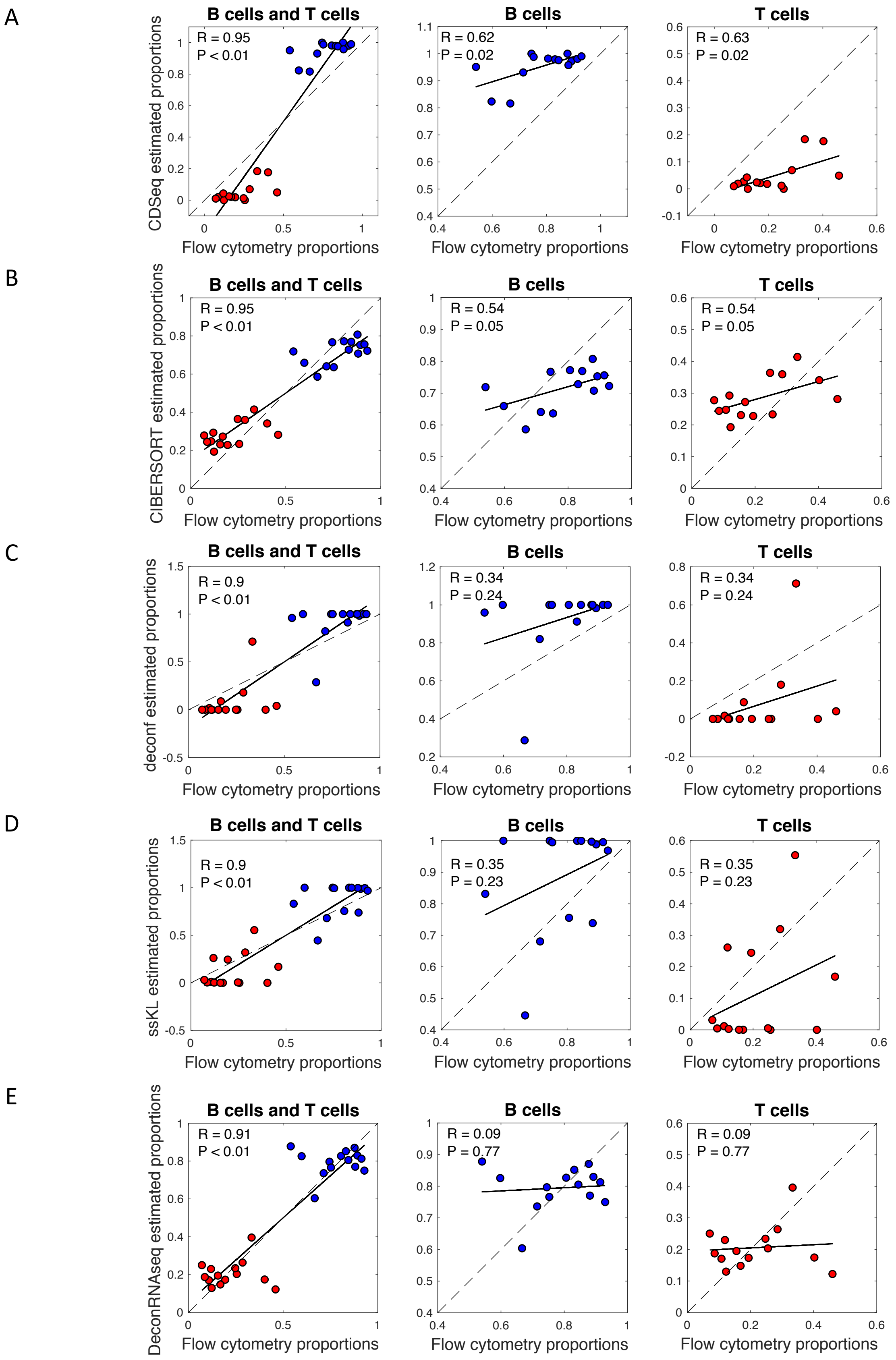

A
B cells and T cells
R = 0.95
P < 0.01
B cells
R = 0.62
P = 0.02
T cells
R = 0.63
P = 0.02
CDSeq estimated proportions
Flow cytometry proportions
B
B cells and T cells
R = 0.95
P < 0.01
B cells
R = 0.54
P = 0.05
T cells
R = 0.54
P = 0.05
CIBERSORT estimated proportions
Flow cytometry proportions
C
B cells and T cells
R = 0.9
P < 0.01
B cells
R = 0.34
P = 0.24
T cells
R = 0.34
P = 0.24
deconf estimated proportions
Flow cytometry proportions
D
B cells and T cells
R = 0.9
P < 0.01
B cells
R = 0.35
P = 0.23
T cells
R = 0.35
P = 0.23
ssKL estimated proportions
Flow cytometry proportions
E
B cells and T cells
R = 0.91
P < 0.01
B cells
R = 0.09
P = 0.77
T cells
R = 0.09
P = 0.77
DeconRNAseq estimated proportions
Flow cytometry proportions

F

Correlations between deconf estimated csGEPs and GEPs of leukocyte subtype

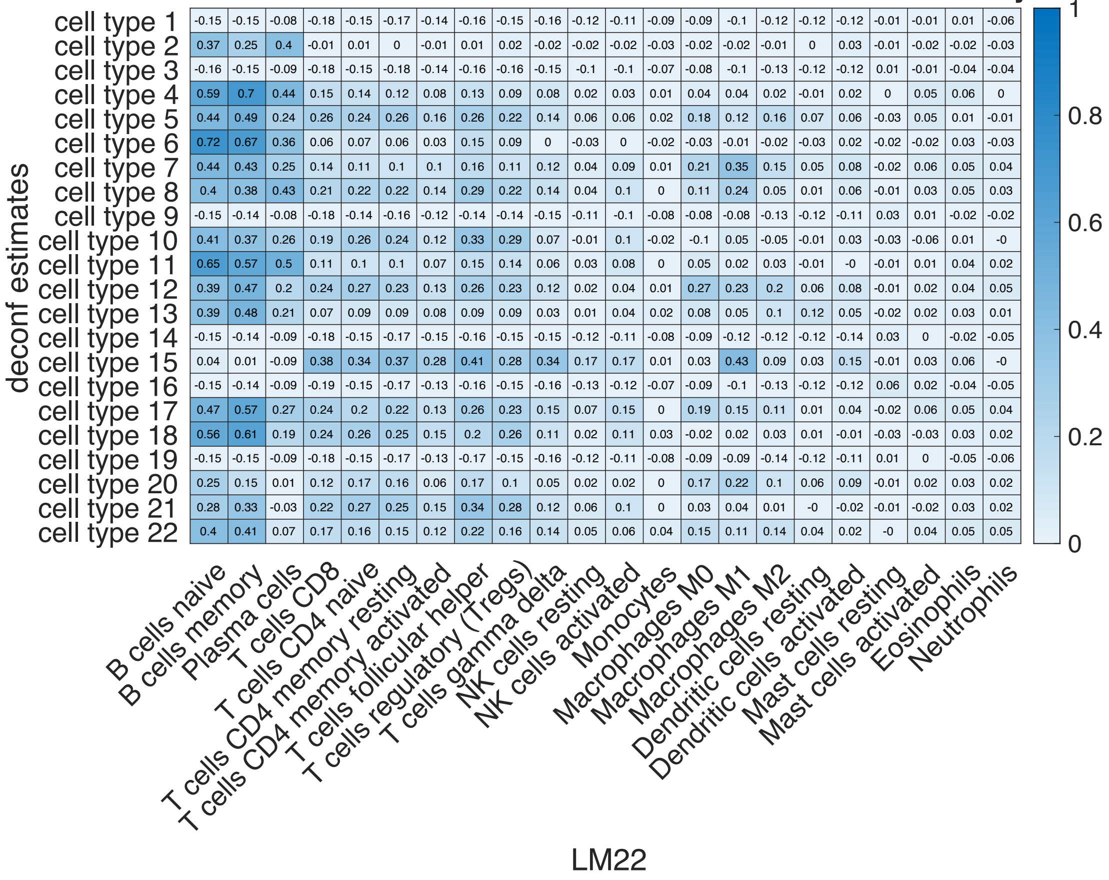

G

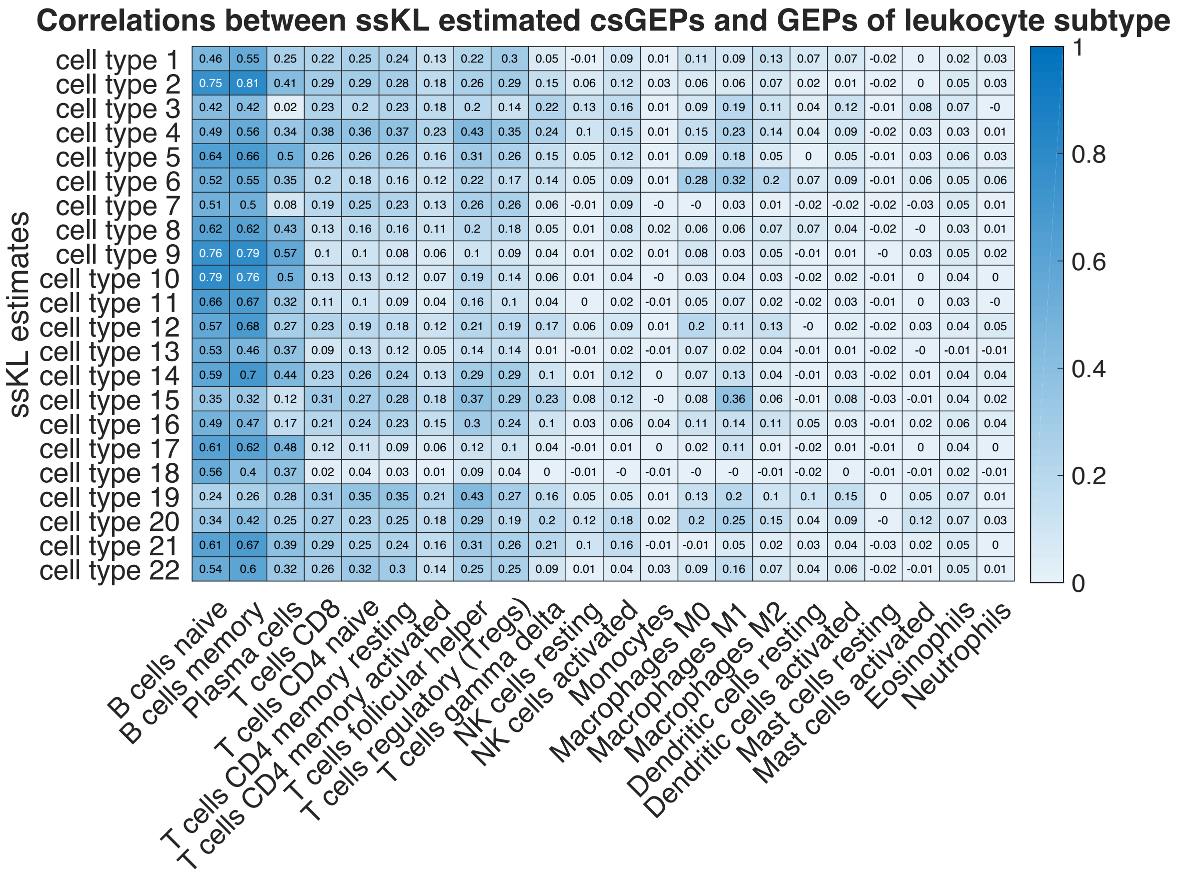

H

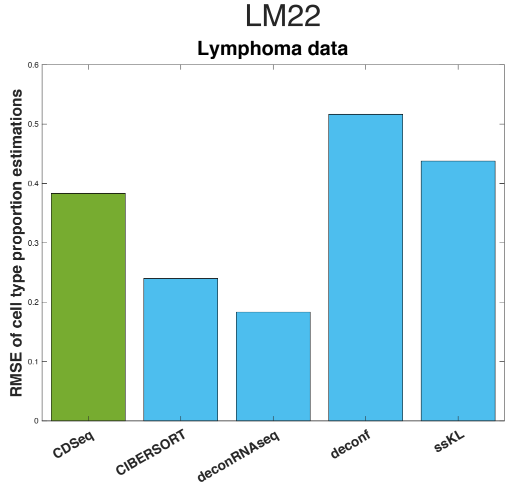

S7 Fig. Results of deconvolution of 14 follicular lymphoma tumors samples. We show flow-cytometry measurements versus estimated cell proportions: (A) CDSeq; (B) CIBERSORT; (C) DeconRNAseq; (D) deconf; (E) ssKL. We used 0.6 as a threshold to infer the identities of estimated cell types. We show correlations between estimated csGEPs and the LM22 GEPs: (F) deconf; (G) ssKL; (H) RMSEs of proportion estimations. For deconf and ssKL, we used the cell type with the highest correlation (0.38 and 0.35, respectively) with T cell GEPs as the T cell population.
