# Supplementary material for: CDSeq: A novel complete deconvolution method for dissecting heterogeneous samples using gene expression data
Source: PLoS Comput Biol. 2019 Dec 2;15(12):e1007510. doi: 10.1371/journal.pcbi.1007510 (PMC6907860; doi:10.1371/journal.pcbi.1007510)
Supplement: S8 Fig — (PDF) [file pcbi.1007510.s011.pdf]

CDSeq on deep deconvolution

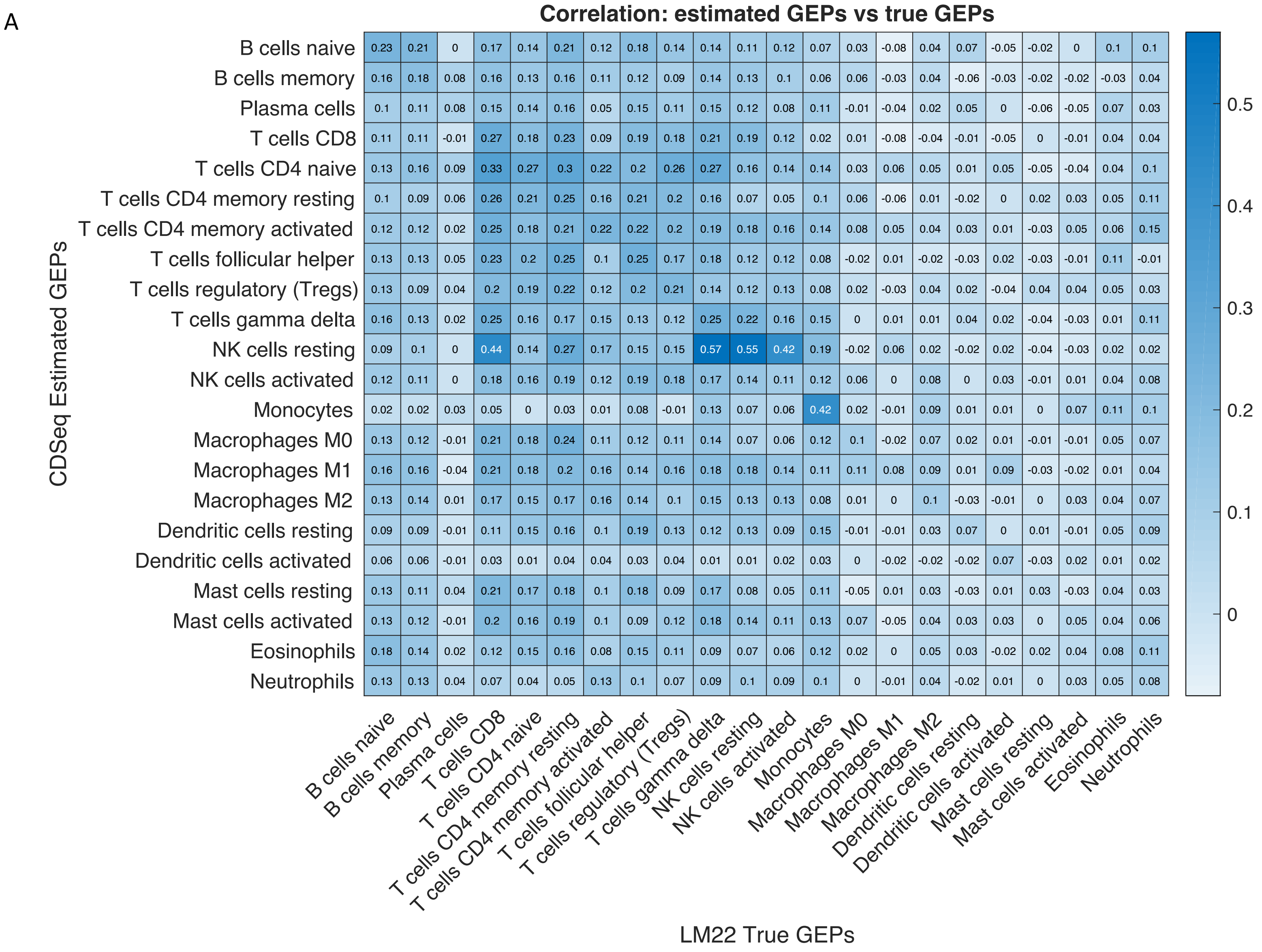

B

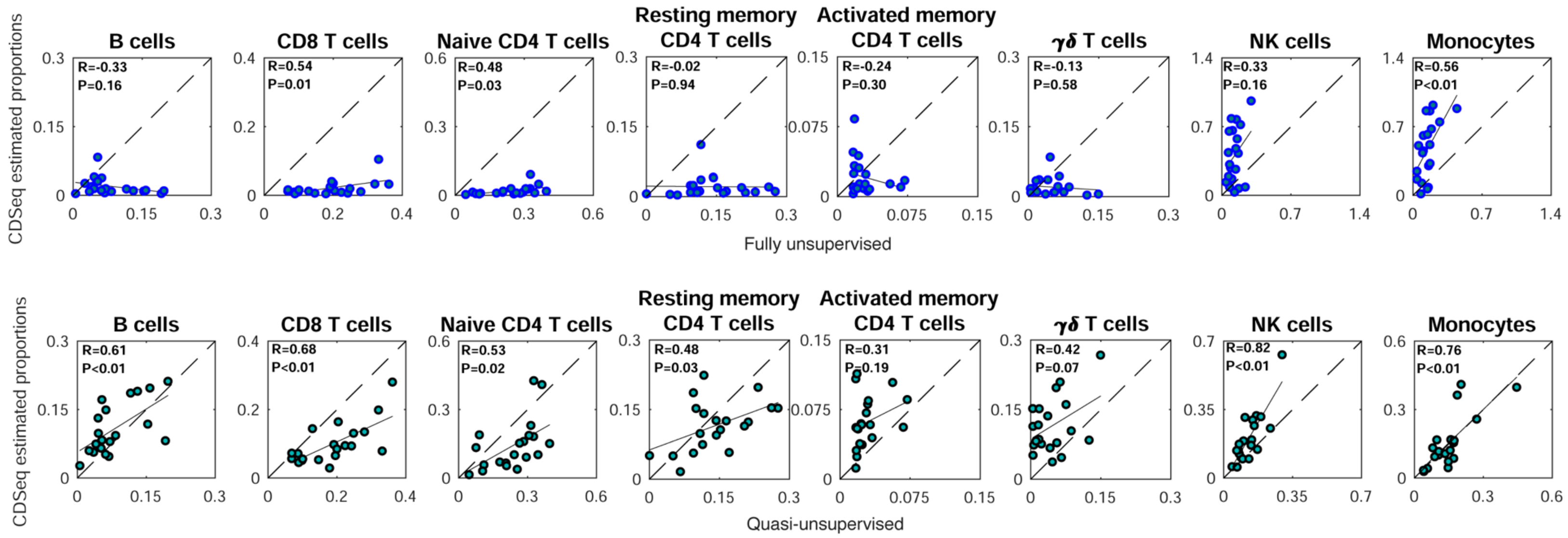
B cells
R=-0.33
P=0.16
CD8 T cells
R=0.54
P=0.01
Naive CD4 T cells
R=0.48
P=0.03
Resting memory CD4 T cells
R=-0.02
P=0.94
Activated memory CD4 T cells
R=-0.24
P=0.30
γδ T cells
R=-0.13
P=0.58
NK cells
R=0.33
P=0.16
Monocytes
R=0.56
P<0.01
CDSeq estimated proportions
Fully unsupervised
B cells
R=0.61
P<0.01
CD8 T cells
R=0.68
P<0.01
Naive CD4 T cells
R=0.53
P=0.02
Resting memory CD4 T cells
R=0.48
P=0.03
Activated memory CD4 T cells
R=0.31
P=0.19
γδ T cells
R=0.42
P=0.07
NK cells
R=0.82
P<0.01
Monocytes
R=0.76
P<0.01
CDSeq estimated proportions
Quasi-unsupervised

C

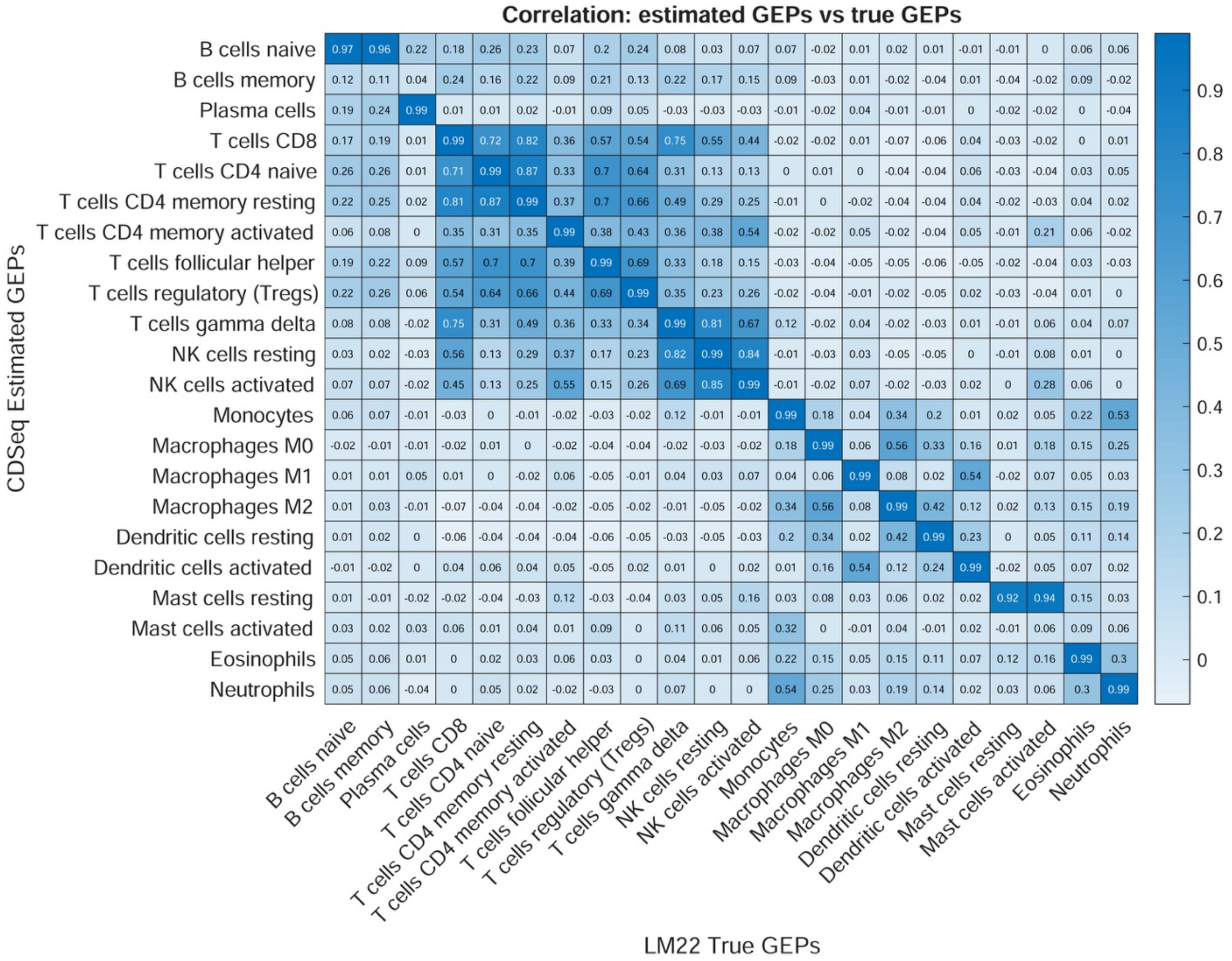
Correlation: estimated GEPs vs true GEPs
CDSeq Estimated GEPs
LM22 True GEPs
B cells naive 0.97 0.96 0.22 0.18 0.26 0.23 0.07 0.2 0.24 0.08 0.03 0.07 0.07 -0.02 0.01 0.02 0.01 -0.01 -0.01 0 0.06 0.06
B cells memory 0.12 0.11 0.04 0.24 0.16 0.22 0.09 0.21 0.13 0.22 0.17 0.15 0.09 -0.03 0.01 -0.02 -0.04 0.01 -0.04 -0.02 0.09 -0.02
Plasma cells 0.19 0.24 0.99 0.01 0.01 0.02 -0.01 0.09 0.05 -0.03 -0.03 -0.03 -0.01 -0.02 0.04 -0.01 -0.01 0 -0.02 -0.02 0 -0.04
T cells CD8 0.17 0.19 0.01 0.99 0.72 0.82 0.36 0.57 0.54 0.75 0.55 0.44 -0.02 -0.02 0.01 -0.07 -0.06 0.04 -0.03 -0.02 0 0.01
T cells CD4 naive 0.26 0.26 0.01 0.71 0.99 0.87 0.33 0.7 0.64 0.31 0.13 0.13 0 0.01 0 -0.04 -0.04 0.06 -0.03 -0.04 0.03 0.05
T cells CD4 memory resting 0.22 0.25 0.02 0.81 0.87 0.99 0.37 0.7 0.66 0.49 0.29 0.25 -0.01 0 -0.02 -0.04 -0.04 0.04 -0.02 -0.03 0.04 0.02
T cells CD4 memory activated 0.06 0.08 0 0.35 0.31 0.35 0.99 0.38 0.43 0.36 0.38 0.54 -0.02 -0.02 0.05 -0.02 -0.04 0.05 -0.01 0.21 0.06 -0.02
T cells follicular helper 0.19 0.22 0.09 0.57 0.7 0.7 0.39 0.99 0.69 0.33 0.18 0.15 -0.03 -0.04 -0.05 -0.05 -0.06 -0.05 -0.02 -0.04 0.03 -0.03
T cells regulatory (Tregs) 0.22 0.26 0.06 0.54 0.64 0.66 0.44 0.69 0.99 0.35 0.23 0.26 -0.02 -0.04 -0.01 -0.02 -0.05 0.02 -0.03 -0.04 0.01 0
T cells gamma delta 0.08 0.08 -0.02 0.75 0.31 0.49 0.36 0.33 0.34 0.99 0.81 0.67 0.12 -0.02 0.04 -0.02 -0.03 0.01 -0.01 0.06 0.04 0.07
NK cells resting 0.03 0.02 -0.03 0.56 0.13 0.29 0.37 0.17 0.23 0.82 0.99 0.84 -0.01 -0.03 0.03 -0.05 -0.05 0 -0.01 0.08 0.01 0
NK cells activated 0.07 0.07 -0.02 0.45 0.13 0.25 0.55 0.15 0.26 0.69 0.85 0.99 -0.01 -0.02 0.07 -0.02 -0.03 0.02 0 0.28 0.06 0
Monocytes 0.06 0.07 -0.01 -0.03 0 -0.01 -0.02 -0.03 -0.02 0.12 -0.01 -0.01 0.99 0.18 0.04 0.34 0.2 0.01 0.02 0.05 0.22 0.53
Macrophages M0 -0.02 -0.01 -0.01 -0.02 0.01 0 -0.02 -0.04 -0.04 -0.02 -0.03 -0.02 0.18 0.99 0.06 0.56 0.33 0.16 0.01 0.18 0.15 0.25
Macrophages M1 0.01 0.01 0.05 0.01 0 -0.02 0.06 -0.05 -0.01 0.04 0.03 0.07 0.04 0.06 0.99 0.08 0.02 0.54 -0.02 0.07 0.05 0.03
Macrophages M2 0.01 0.03 -0.01 -0.07 -0.04 -0.04 -0.02 -0.05 -0.02 -0.01 -0.05 -0.02 0.34 0.56 0.08 0.99 0.42 0.12 0.02 0.13 0.15 0.19
Dendritic cells resting 0.01 0.02 0 -0.06 -0.04 -0.04 -0.04 -0.06 -0.05 -0.03 -0.05 -0.03 0.2 0.34 0.02 0.42 0.99 0.23 0 0.05 0.11 0.14
Dendritic cells activated -0.01 -0.02 0 0.04 0.06 0.04 0.05 -0.05 0.02 0.01 0 0.02 0.01 0.16 0.54 0.12 0.24 0.99 -0.02 0.05 0.07 0.02
Mast cells resting 0.01 -0.01 -0.02 -0.02 -0.04 -0.03 0.12 -0.03 -0.04 0.03 0.05 0.16 0.03 0.08 0.03 0.06 0.02 0.02 0.92 0.94 0.15 0.03
Mast cells activated 0.03 0.02 0.03 0.06 0.01 0.04 0.01 0.09 0 0.11 0.06 0.05 0.32 0 -0.01 0.04 -0.01 0.02 -0.01 0.06 0.09 0.06
Eosinophils 0.05 0.06 0.01 0 0.02 0.03 0.06 0.03 0 0.04 0.01 0.06 0.22 0.15 0.05 0.15 0.11 0.07 0.12 0.16 0.99 0.3
Neutrophils 0.05 0.06 -0.04 0 0.05 0.02 -0.02 -0.03 0 0.07 0 0 0.54 0.25 0.03 0.19 0.14 0.02 0.03 0.06 0.3 0.99
B cells naive
B cells memory
Plasma cells
T cells CD8
T cells CD4 naive
T cells CD4 memory resting
T cells CD4 memory activated
T cells follicular helper
T cells regulatory (Tregs)
T cells gamma delta
NK cells resting
NK cells activated
Monocytes
Macrophages M0
Macrophages M1
Macrophages M2
Dendritic cells resting
Dendritic cells activated
Mast cells resting
Mast cells activated
Eosinophils
Neutrophils
0.9
0.8
0.7
0.6
0.5
0.4
0.3
0.2
0.1
0

D

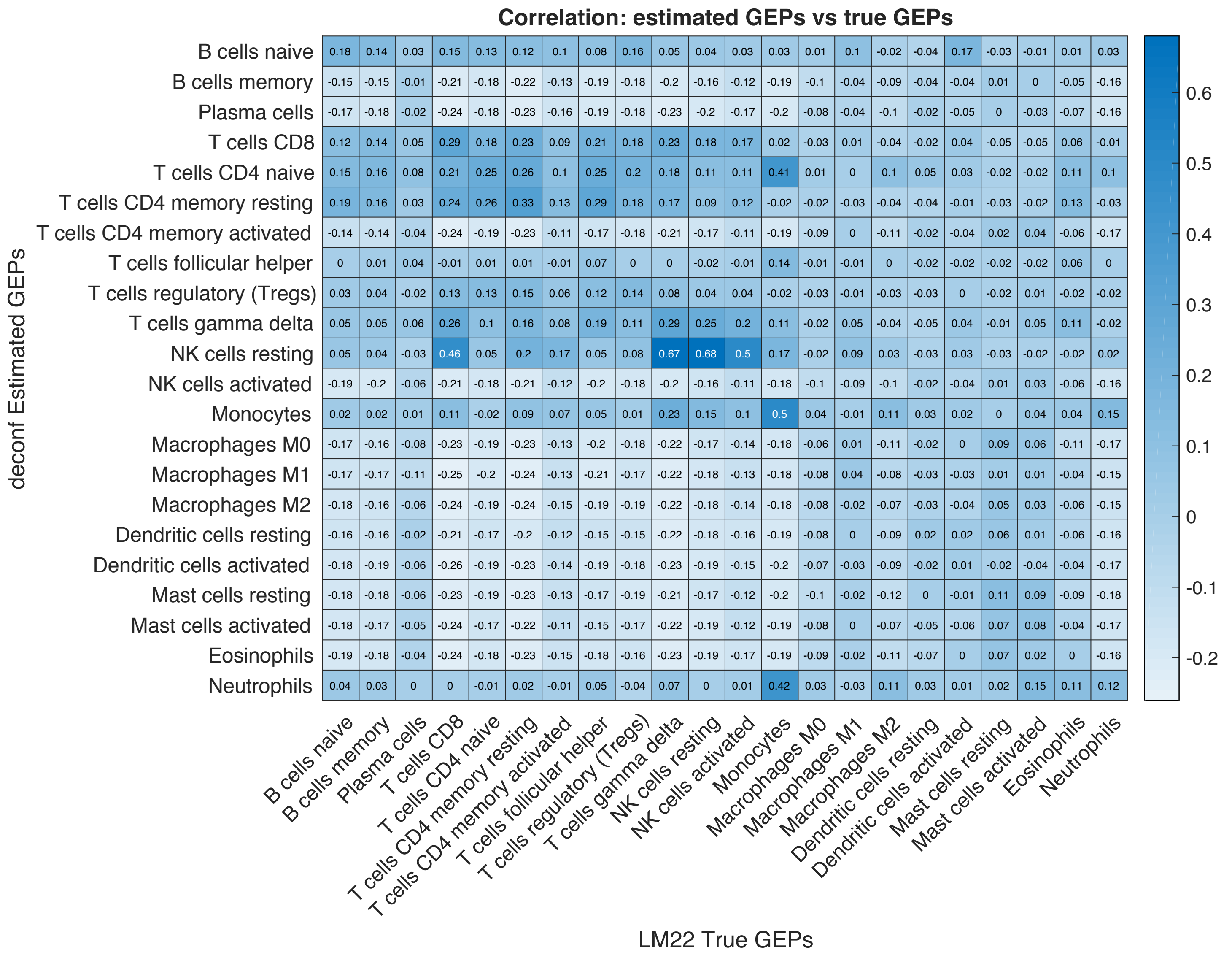
Correlation: estimated GEPs vs true GEPs
deconf Estimated GEPs
LM22 True GEPs
B cells naive
B cells memory
Plasma cells
T cells CD8
T cells CD4 naive
T cells CD4 memory resting
T cells CD4 memory activated
T cells follicular helper
T cells regulatory (Tregs)
T cells gamma delta
NK cells resting
NK cells activated
Monocytes
Macrophages M0
Macrophages M1
Macrophages M2
Dendritic cells resting
Dendritic cells activated
Mast cells resting
Mast cells activated
Eosinophils
Neutrophils
0.6
0.5
0.4
0.3
0.2
0.1
0
-0.1
-0.2

E

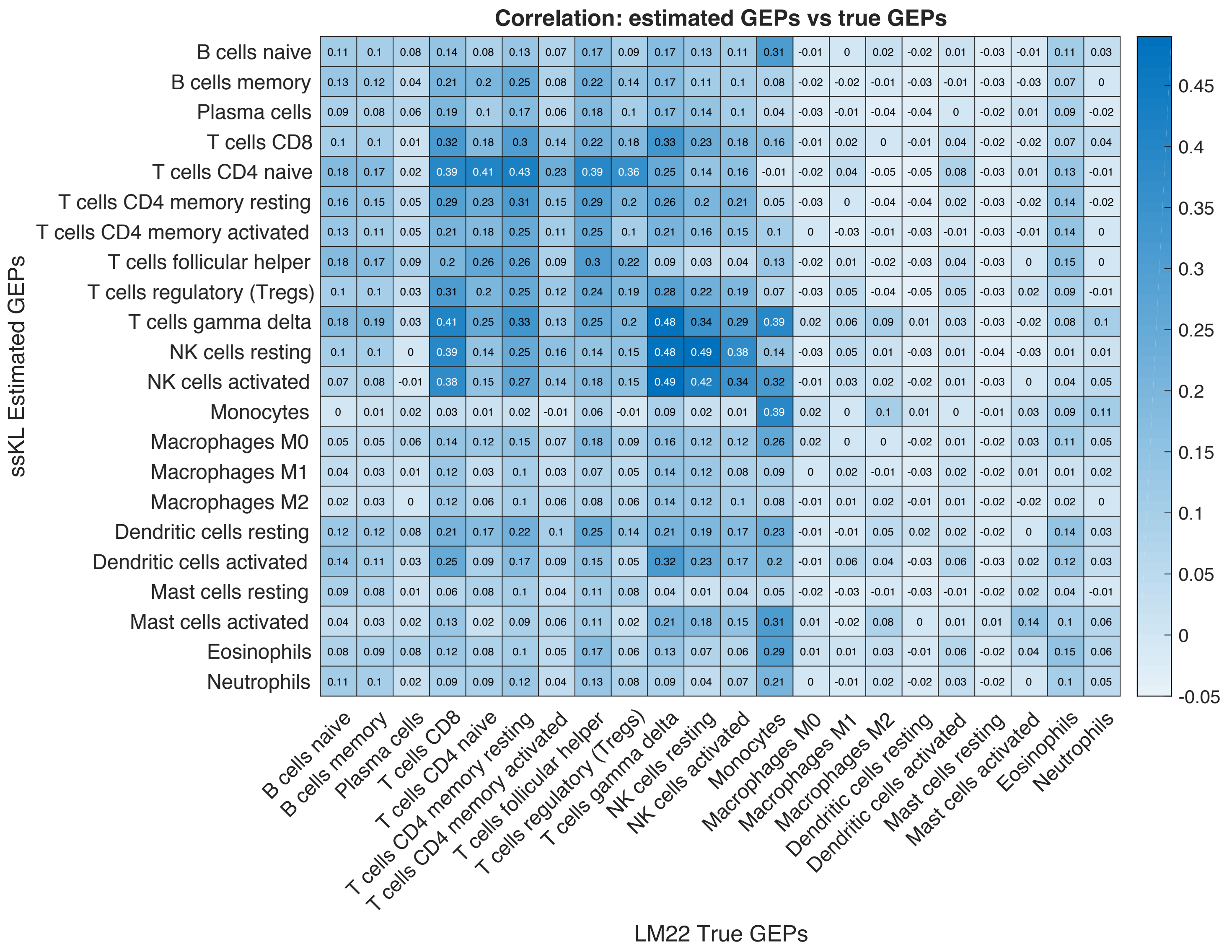
Correlation: estimated GEPs vs true GEPs
ssKL Estimated GEPs
LM22 True GEPs
B cells naive
B cells memory
Plasma cells
T cells CD8
T cells CD4 naive
T cells CD4 memory resting
T cells CD4 memory activated
T cells follicular helper
T cells regulatory (Tregs)
T cells gamma delta
NK cells resting
NK cells activated
Monocytes
Macrophages M0
Macrophages M1
Macrophages M2
Dendritic cells resting
Dendritic cells activated
Mast cells resting
Mast cells activated
Eosinophils
Neutrophils
0.45
0.4
0.35
0.3
0.25
0.2
0.15
0.1
0.05
0
-0.05

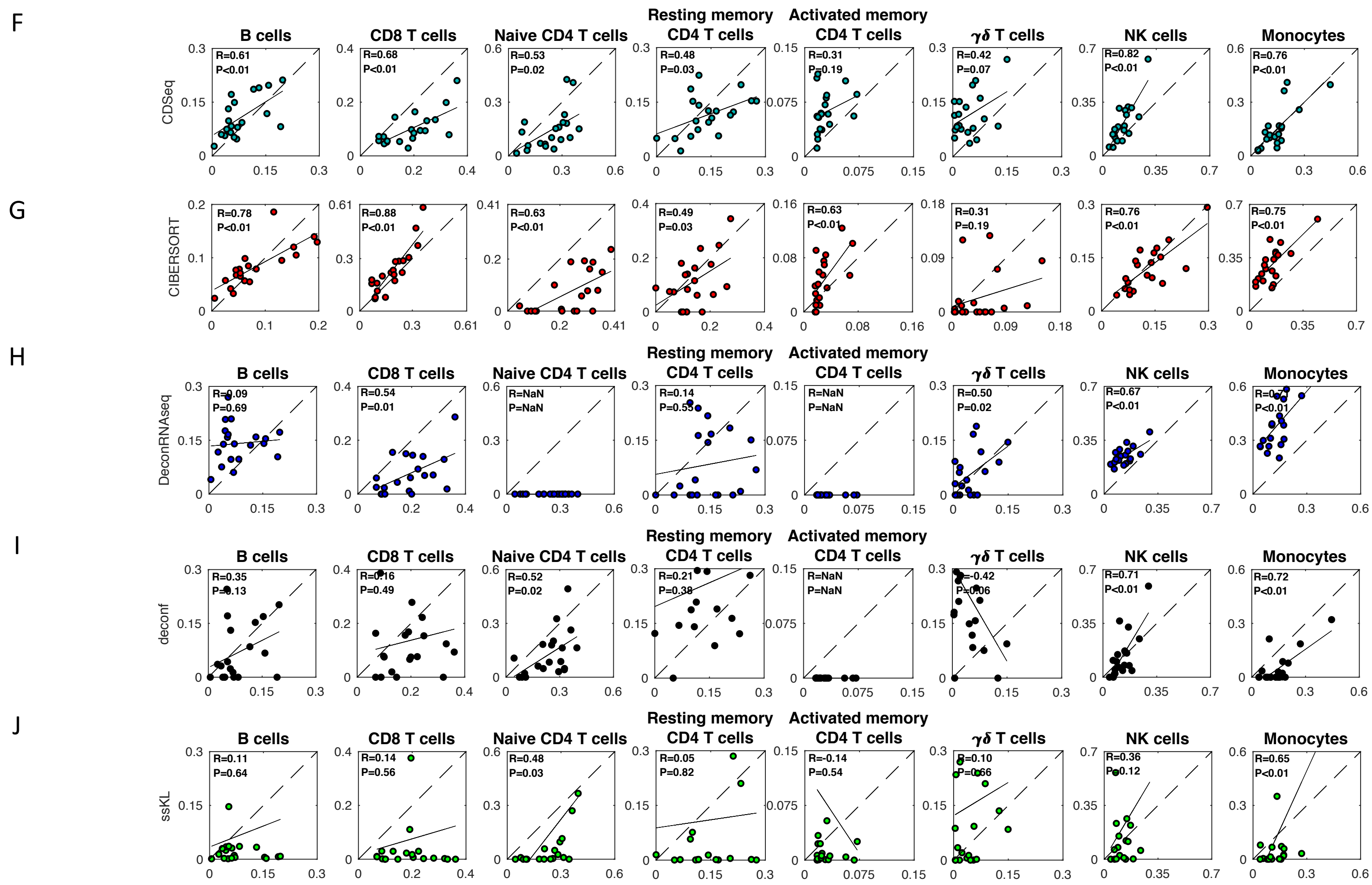
F
G
H
I
J
B cells
CD8 T cells
Naive CD4 T cells
Resting memory CD4 T cells
Activated memory CD4 T cells
γδ T cells
NK cells
Monocytes
CDSeq
CIBERSORT
DeconRNAseq
deconf
ssKL
R=0.61 P<0.01
R=0.68 P<0.01
R=0.53 P=0.02
R=0.48 P=0.03
R=0.31 P=0.19
R=0.42 P=0.07
R=0.82 P<0.01
R=0.76 P<0.01
R=0.78 P<0.01
R=0.88 P<0.01
R=0.63 P<0.01
R=0.49 P=0.03
R=0.63 P<0.01
R=0.31 P=0.19
R=0.76 P<0.01
R=0.75 P<0.01
R=0.09 P=0.69
R=0.54 P=0.01
R=NaN P=NaN
R=0.14 P=0.55
R=NaN P=NaN
R=0.50 P=0.02
R=0.67 P<0.01
R=0.35 P=0.13
R=0.16 P=0.49
R=0.52 P=0.02
R=0.21 P=0.38
R=NaN P=NaN
R=-0.42 P=0.06
R=0.71 P<0.01
R=0.72 P<0.01
R=0.11 P=0.64
R=0.14 P=0.56
R=0.48 P=0.03
R=0.05 P=0.82
R=-0.14 P=0.54
R=0.10 P=0.66
R=0.36 P=0.12
R=0.65 P<0.01

K

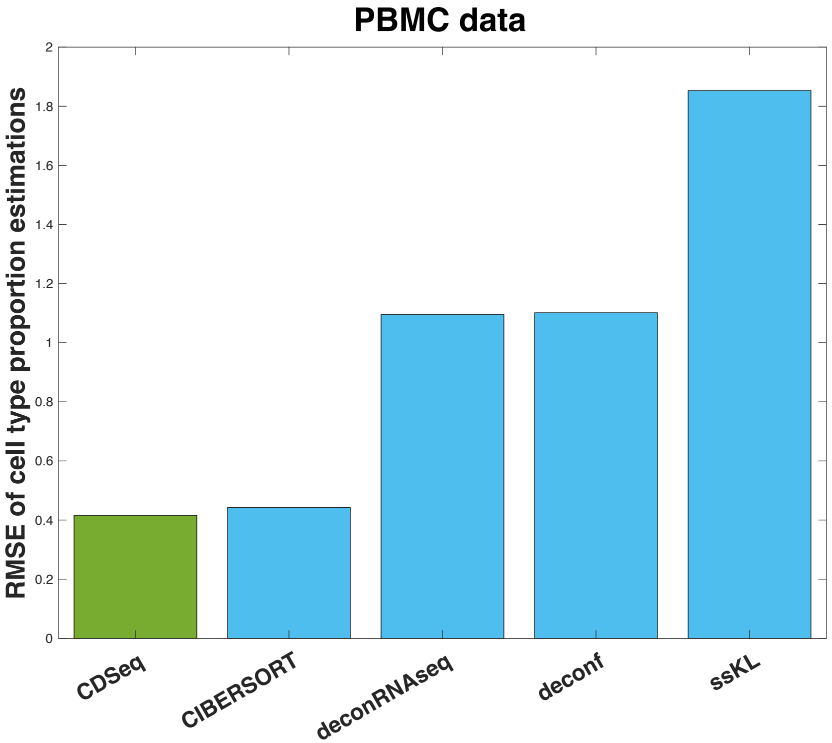

S8 Fig. Performance comparison on deep deconvolution. We applied CDSeq, CIBERSORT, DeconRNAseq, deconf, ssKL on PBMC data set. We set the number of cell types to be 22, α=50, β=20 for CDSeq and used default settings for other methods. R is the correlation coefficient; and P is the p-value for testing the null hypothesis of no correlation: (A) Heat map of correlations between CDSeq-estimated cell-type-specific GEPs and LM22 GEPs; and, (B) CDSeq-estimated cell-type proportions compared to flow cytometry estimates, where the upper panel (blue dots) is the result of fully unsupervised mode and the lower panel (green dots) is the result of quasi-unsupervised mode. The black line is the linear regression line; We show correlations between estimated cell-type-specific GEPs and LM22 GEPs by different methods: (C) CDSeq with quasi-unsupervised learning (heatmap of correlations of true GEPs is given in **S5 Fig.**); (D) deconf; (E) ssKL; We show estimated SSP versus flow-cytometry measurements by different methods: (F) CDSeq with quasi-unsupervised learning; (G) CIBERSORT; (H) DeconRNAseq; (I) deconf; (J) ssKL; (K) RMSEs of cell type proportion estimations.
