# Supplementary material for: CDSeq: A novel complete deconvolution method for dissecting heterogeneous samples using gene expression data
Source: PLoS Comput Biol. 2019 Dec 2;15(12):e1007510. doi: 10.1371/journal.pcbi.1007510 (PMC6907860; doi:10.1371/journal.pcbi.1007510)
Supplement: S9 Fig — (PDF) [file pcbi.1007510.s012.pdf]

Effect of dilution factor on performance

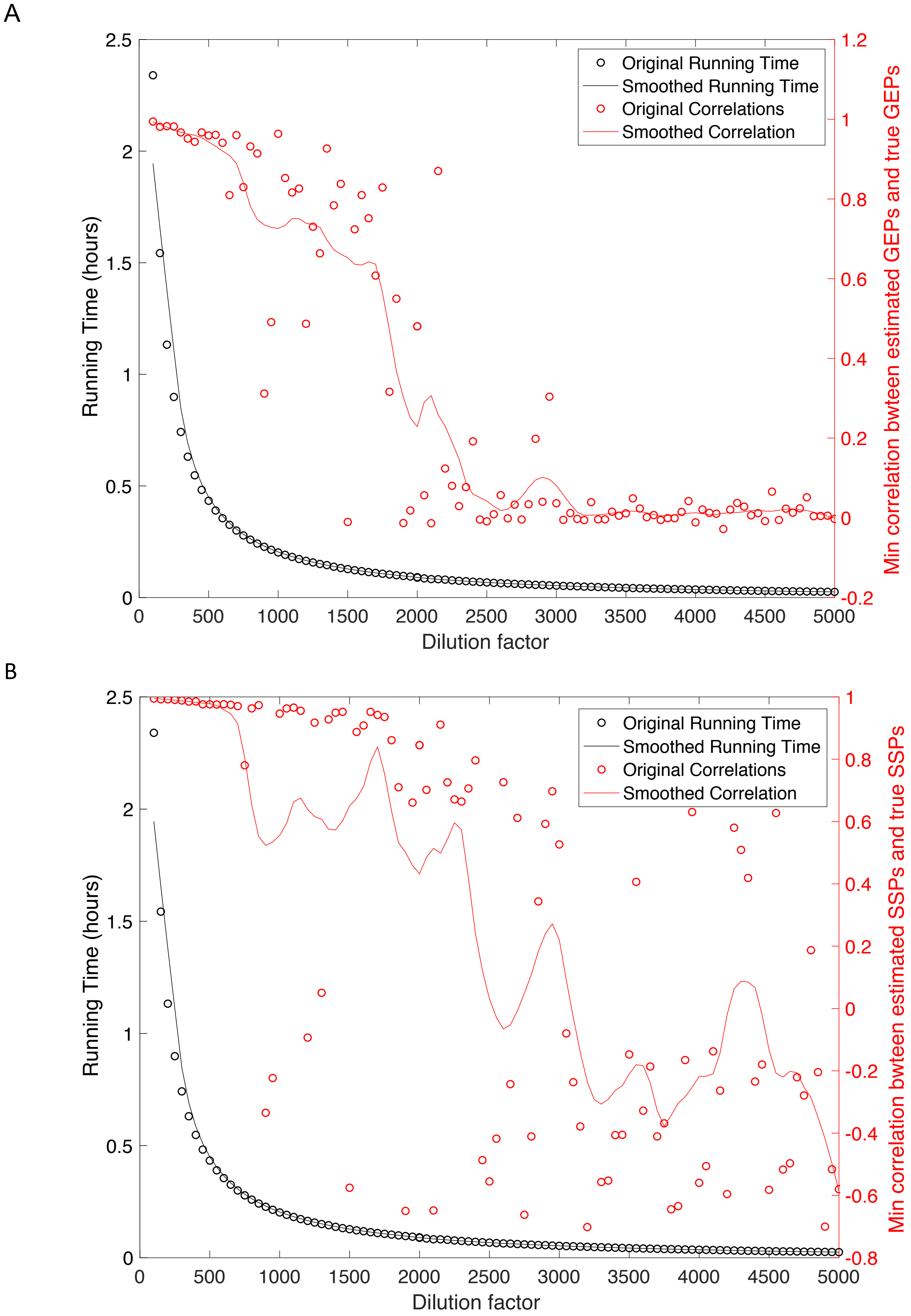

C

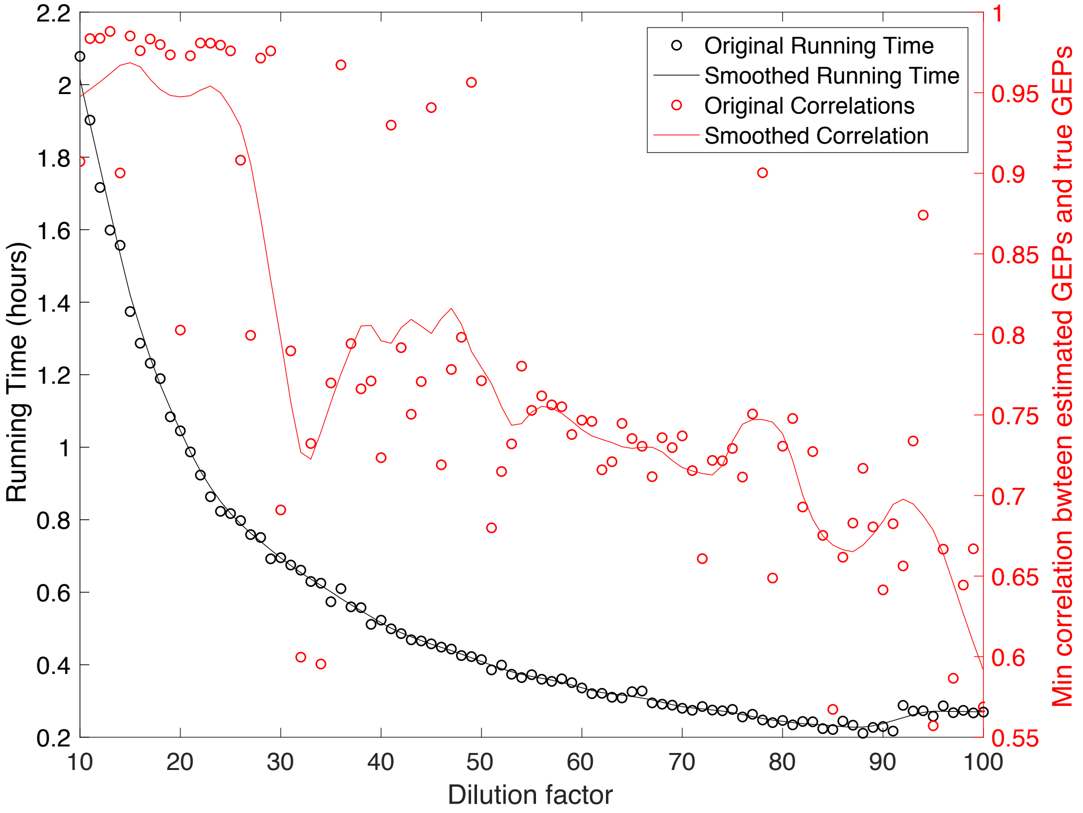

Original Running Time
Smoothed Running Time
Original Correlations
Smoothed Correlation
Running Time (hours)
Min correlation bwteen estimated GEPs and true GEPs
Dilution factor

D

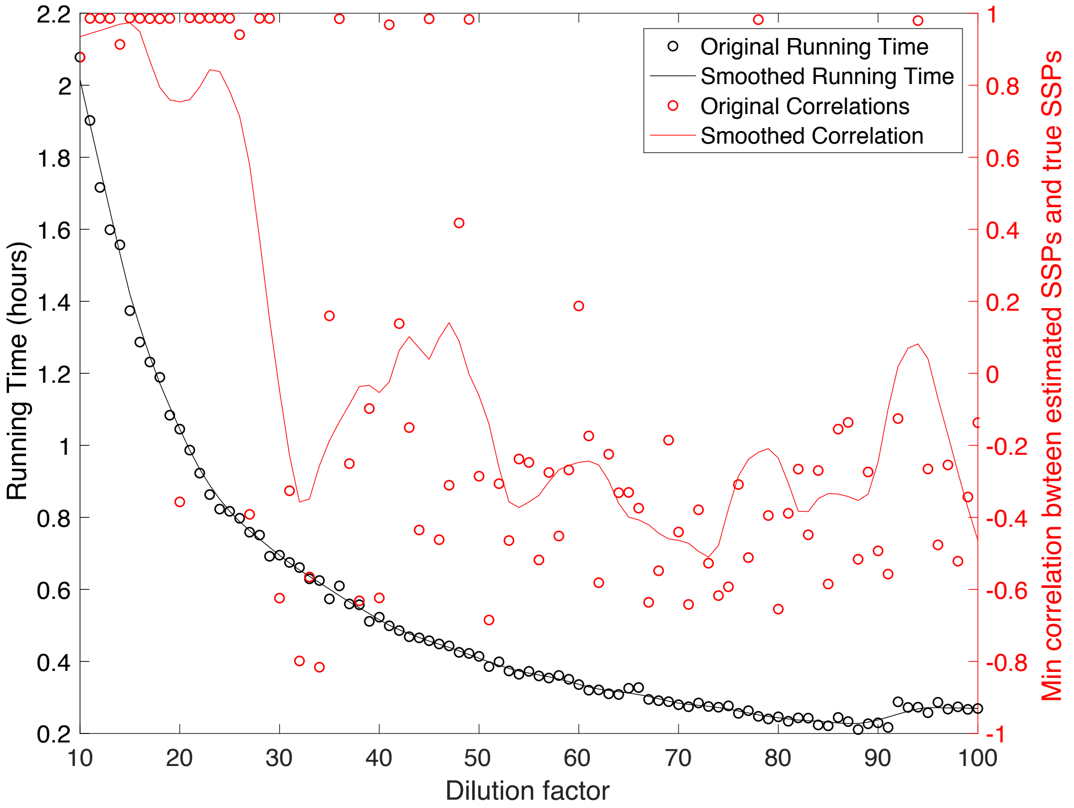

Original Running Time
Smoothed Running Time
Original Correlations
Smoothed Correlation
Running Time (hours)
Min correlation bwteen estimated SSPs and true SSPs
Dilution factor

S9 Fig. Running time of CDSeq plotted against the dilution factor for data dilution using the synthetic data and experimental data. Data dilution is a strategy to avoid large memory cost and a way to speed up calculations. One divides the read counts by a constant dilution factor. For varying dilution factors, we measured the running time (in hours) and recorded the minimum correlations between the six CDSeq-identified csGEPs and their matched (using Munkres algorithm) six true GEPs and the minimum correlations between estimated SSP and true SSP: (A, B). synthetic data; (C, D). experimental data. As the dilution factor increases, the running time decreases rapidly whereas the minimum of the Pearson correlation coefficients decreases more slowly and reaches a plateau near zero, indicating that at least one of the true cell types could not be identified, after dilution factors exceed about 2000. In this example, the total number of reads for all the 40 synthetic samples is about 9.5×10^9. We used dilution factors ranging from 100 to 1000 in increments of 50 for synthetic data and from 10 to 100 with increments of 1 for experimental data. For experimental data, the range of dilution factors used are much smaller than those of synthetic data. This is because the sequencing depth of the pure cell lines used for creating the synthetic samples are about 10 times higher than those of experimental data. The total reads for all 32 experimental samples are about 6×10^8. In addition, there is no technical noise in those *in silico* Mixtures.
