# Supplementary material for: CDSeq: A novel complete deconvolution method for dissecting heterogeneous samples using gene expression data
Source: PLoS Comput Biol. 2019 Dec 2;15(12):e1007510. doi: 10.1371/journal.pcbi.1007510 (PMC6907860; doi:10.1371/journal.pcbi.1007510)
Supplement: S10 Fig — (PDF) [file pcbi.1007510.s013.pdf]

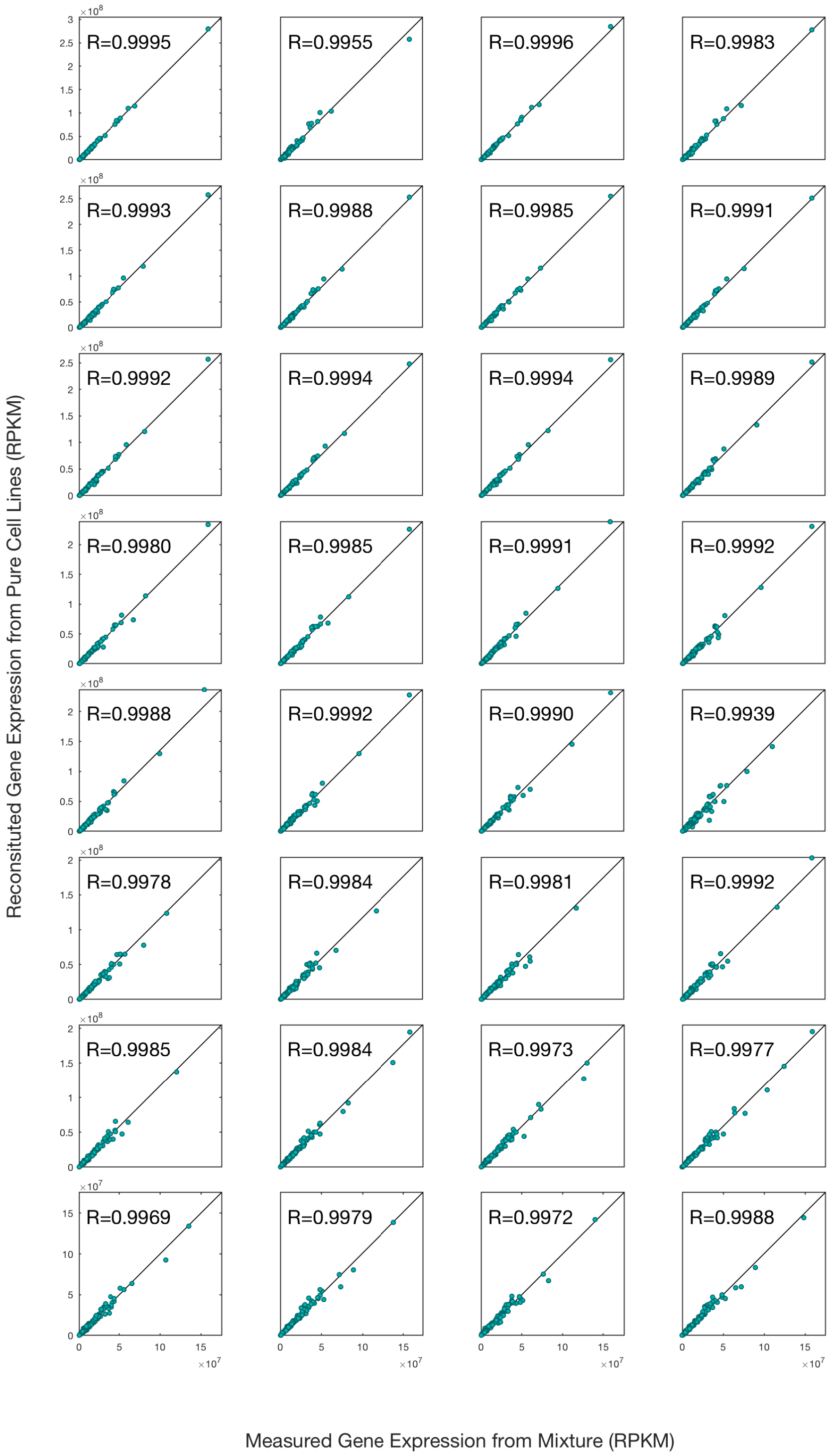

S10 Fig. Linearity assumption test using 32 experimental mixtures. We tested the linearity assumption employed by csSAM and other deconvolution methods by comparing the measured RPKMs with the reconstituted RPKMs using known mixing proportions and four pure cell lines. *x*-axis represents the measured GEP of mixtures (in RPKM) and the y-axis denotes the RPKMs of the reconstituted mixtures. *R* denotes the correlation between measured mixtures RPKM and the reconstituted RPKM.
